# Stochastic Measurement Models for Quantifying Lymphocyte Responses Using Flow Cytometry

**DOI:** 10.1371/journal.pone.0146227

**Published:** 2016-01-07

**Authors:** Andrey Kan, Damian Pavlyshyn, John F. Markham, Mark R. Dowling, Susanne Heinzel, Jie H. S. Zhou, Julia M. Marchingo, Philip D. Hodgkin

**Affiliations:** 1 Division of Immunology, The Walter and Eliza Hall Institute of Medical Research, Parkville, VIC, Australia; 2 Department of Medical Biology, The University of Melbourne, Parkville, VIC, Australia; University of Glasgow, UNITED KINGDOM

## Abstract

Adaptive immune responses are complex dynamic processes whereby B and T cells undergo division and differentiation triggered by pathogenic stimuli. Deregulation of the response can lead to severe consequences for the host organism ranging from immune deficiencies to autoimmunity. Tracking cell division and differentiation by flow cytometry using fluorescent probes is a major method for measuring progression of lymphocyte responses, both in vitro and in vivo. In turn, mathematical modeling of cell numbers derived from such measurements has led to significant biological discoveries, and plays an increasingly important role in lymphocyte research. Fitting an appropriate parameterized model to such data is the goal of these studies but significant challenges are presented by the variability in measurements. This variation results from the sum of experimental noise and intrinsic probabilistic differences in cells and is difficult to characterize analytically. Current model fitting methods adopt different simplifying assumptions to describe the distribution of such measurements and these assumptions have not been tested directly. To help inform the choice and application of appropriate methods of model fitting to such data we studied the errors associated with flow cytometry measurements from a wide variety of experiments. We found that the mean and variance of the noise were related by a power law with an exponent between 1.3 and 1.8 for different datasets. This violated the assumptions inherent to commonly used least squares, linear variance scaling and log-transformation based methods. As a result of these findings we propose a new measurement model that we justify both theoretically, from the maximum entropy standpoint, and empirically using collected data. Our evaluation suggests that the new model can be reliably used for model fitting across a variety of conditions. Our work provides a foundation for modeling measurements in flow cytometry experiments thus facilitating progress in quantitative studies of lymphocyte responses.

## Introduction

In response to pathogenic stimuli, B and T lymphocytes undergo proliferation and differentiation into effector and memory cells. Dysregulation at any stage of this process can lead to severe consequences for the host organism, ranging from autoimmune diseases and transplant rejection to immunodeficiency and cancer. Obtaining a quantitative understanding of lymphocyte response regulation poses a significant theoretical and practical challenge for modern immunology. It is increasingly evident that the study of such a complex biological phenomenon will require an interdisciplinary approach. Indeed, mathematical modeling of proliferating lymphocytes has played a central role in a number of major biological discoveries [1–3]. Moreover, a quantitative study of the effects of different signals on proliferation and survival parameters has enabled accurate prediction of lymphocyte expansion kinetics in response to signal modulation [4]. Such predictive power can be employed in next generation drug screening platforms for a range of therapies, including cancer immunotherapy [5].

Flow cytometry using a wide array of fluorescence probes is one of the most powerful methods used to measure the progression of lymphocyte responses both *in vivo* and *in vitro* [6]. It is therefore not surprising that an increasing number of research groups fit and validate their mathematical models of lymphocyte responses against flow cytometry data [4, 7–13]. Division tracking dyes, such as carboxyfluorescein succinimidyl ester (CFSE) or CellTrace Violet (CTV), enable estimation of the number of cells that have undergone a certain number of divisions after activation [14, 15]. These data alone have been instrumental for developing models of lymphocyte proliferation, such as systems of ordinary differential equations [16], branching process-based formulations [17], or the Cyton model [18].

**Fig 1:**
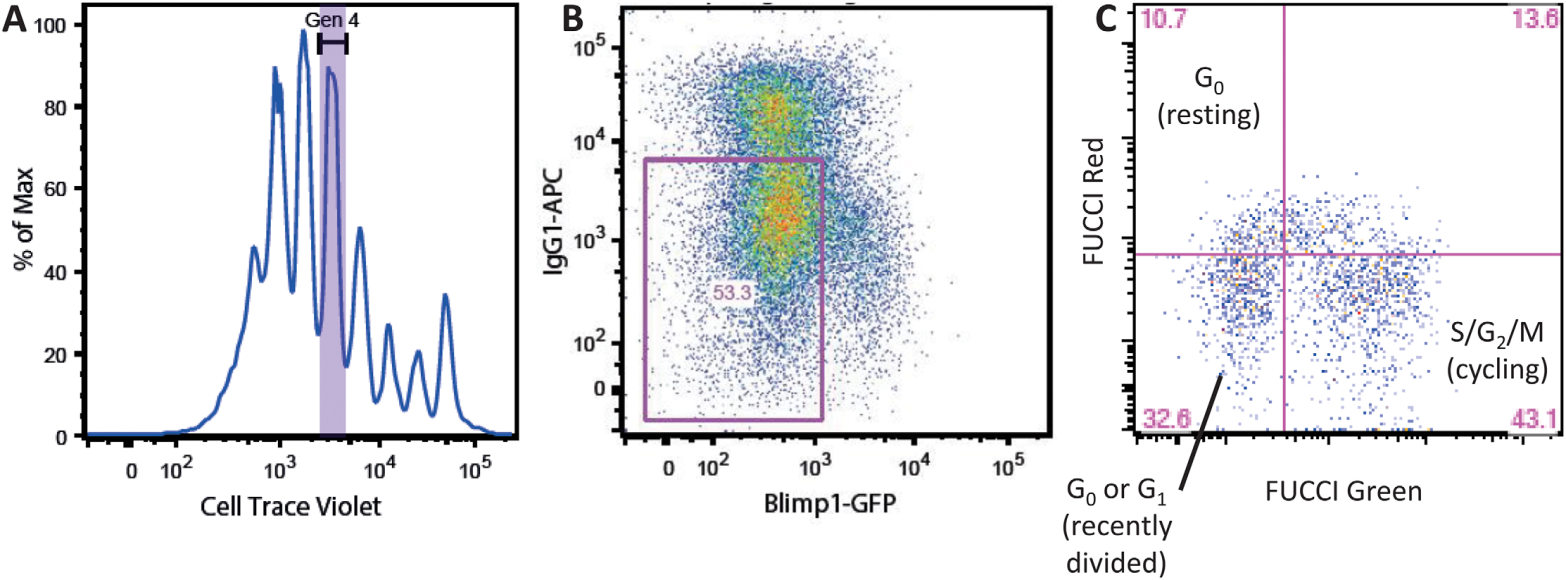
Measuring lymphocyte responses using a range of fluorescent probes. Panels show flow cytometry results for a time point from different representative B or T cell activation experiments. A: CTV can be used to estimate number of cells that divided 4 times since activation (“generation 4” gate). B: Blimp1-GFP reporter and IgG1-APC antibody enable estimation of non-differentiated cells (purple gate). IgG1-APC is a fluorescent probe-conjugated antibody against IgG. C: FUCCI reporter system allows one to estimate cells in different cell cycle stages (labeled quadrants). In each case, estimates are obtained using semi-manual gates. Such gates can be initially set using an automated routine, but it is a common practice to manually validate and adjust the gates.

Moreover, the range of fluorescent probes is not restricted to division tracking (Fig 1). For example, the Blimp1-green fluorescent protein (GFP) reporter mouse is useful for quantifying B cell differentiation into plasma cells [19], and the fluorescent ubiquitination-based cell-cycle indicator (FUCCI) system is a powerful tool for measuring cell cycle progression [20]. These and other types of measurements support the development of mathematical models for various aspects of lymphocyte responses. Here, we consider flow cytometry measurements, obtained using division tracking dyes, or other probes, and the accordingly suitable mathematical models used to fit these data, collectively called *response models*. We note that flow cytometry data is expected to remain a major source of information for the response models in future due to the growing arsenal of fluorescent probes, their applicability *in vivo*, and their broad applicability in many biological contexts.

There is considerable variation in measurements repeated for the same time point and experimental conditions. This variation arises from two sources. First, biological variation arises due to inherent cell-to-cell variability. For example, even sister cells can have different division times or different fates where one cell dies and another cell divides [21]. Second, experimental error denotes collective variations contributed by different steps of the experimental procedure. Experimental error is responsible for the bulk of variation in measurements [22], and therefore cannot be ignored during model fitting. While biological variation can be explicitly characterized as a part of a lymphocyte proliferation model [13, 22], it is extremely difficult to formalize experimental error. This variability originates from pipetting errors, variation in number of cells processed by a flow cytometer (sample recovery), and multi-level semi-manual gating. Note that raw flow cytometry measurements are inevitably subject to at least two rounds of gating, whereby a researcher first separates live cells from calibration beads and dead cells [23], and then applies a gate on a particular fluorescent probe (Fig 1). In some experiments, an additional round of gating is needed to isolate the cell population of interest (e.g., where transgenic cells were adoptively transferred). In practice, all these gates are either drawn entirely manually, or set automatically, but then adjusted manually. As such, experimental error is one of the major challenges of model fitting.

**Fig 2:**
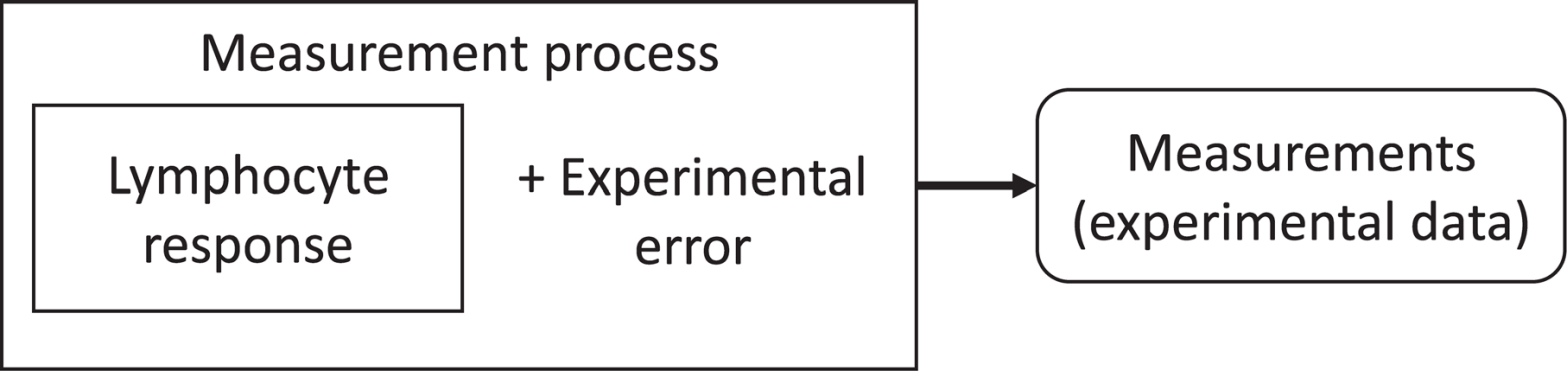
Measurement process encapsulates biological variability and experimental error. Since it is difficult to formalize experimental error, a common strategy is to consider a stochastic measurement generating process as a whole. Measurements can then be described using a measurement distribution. Internally, this distribution is a result of stochastic lymphocyte response and experimental error (including data pre-processing, such as gating).

The difficulty in finding an explicit experimental error characterization has lead to an approach where all the stochastic biological and experimental effects are attributed to a single process (Fig 2) which is formalized using some assumed probability distribution for measurements [10, 11, 13]. This measurement distribution can then be used for model fitting, e.g., assuming normality of measurements with fixed variance leads to least-squares fitting. The problem is that different researchers have been using different sets of assumptions for fitting to the same type of data [10, 11, 13]. Moreover, within the same work one set of assumptions (e.g., measurement variance scaling with the mean) can be adopted for parameter estimation, while another set of assumptions (e.g., constant variance) is used for model selection using information-theoretic criteria [11, 24]. *Here we study the distribution of measurements from representative experiments as an important step towards adoption of a single standard method for estimating parameters from flow cytometry data*.

To this end, we explore lymphocyte response data (both new and previously published) from a wide range of experimental conditions focusing on immunological applications. Based on these data, we develop a new measurement model and corresponding model fitting method. In addition to the data, we provide a theoretical support for our model from a maximum entropy standpoint. Our evaluation shows that the new method can be reliably used for model fitting across a variety of conditions, thus providing a foundation for model development in the field of quantitative lymphocyte research.

## Related Work

There has been a large number of proposed lymphocyte response models, as well as parameter estimation strategies for flow cytometry data [4, 7–13]. In this work, we focus on parameter estimation strategies and the assumptions involved in these strategies. These strategies can be broadly categorized in heuristic and probabilistic approaches. In a heuristic approach, a lymphocyte response model is used to predict expected values of measurements, and an *ad hoc* function is defined to characterize the discrepancy between the predictions and the data [12, 13, 24]. This discrepancy is then minimized as a function of response model parameters. Probabilistic approaches, on the other hand, attempt to define the probability of the measured data given response model parameters [10, 13]. This probability can then be used in different ways, for example, in maximum likelihood (ML) or Bayesian parameter estimation algorithms. It is important to acknowledge that various assumptions can be heuristically adopted in order to derive the probability of measurements. However, the probabilistic approach still has the advantage that all assumptions can be explicitly identified and tested.

A measurement can be viewed as the sum of two random variables corresponding to lymphocyte response and experimental error (Fig 2). In most lymphocyte response models, the population response is considered as the sum of independent single cell responses, and the central limit theorem can be used to argue that the response outcomes are approximately normal. It is then possible to explicitly model experimental error in the context of CFSE experiments by describing the process of peak generation on a CFSE histogram [12, 25]. However, such an approach does not take into account other steps of experimental procedure, including live cell gating, and also is not applicable to other types of flow cytometry experiments, such as those involving Blimp1-GFP or FUCCI reporters. Alternatively, other probabilistic methods assume a certain probability distribution directly for measurements [10, 11, 13, 24]. It is also assumed that expected values of the response model (as a function of model parameters) are indicative of the expected values of the measurements [11, 13, 24]. Next, many methods adopt the simplifying assumption that the measurement covariance matrix is diagonal [10, 11, 24].

Probabilistic measurement models are used for both parameter estimation and model selection based on information criteria, such as corrected Akaike Information Criterion (AICc), and Bayesian Information Criterion (BIC). These criteria require an estimate of the probability of the data [26], i.e., a probabilistic measurement model. For such a model, some authors adopt a multivariate normal distribution with a constant variance [11, 24], while others assume constant marginal variances of log-transformed data [10]. These previous measurement models are formally described in S1 Text. A point of concern here is that in some cases, fitting is performed using a different criterion than the one used for model selection. For example, for the purposes of fitting, marginal variances can be assumed to scale linearly with the expected values, while for the purposes of model selection these variances can be assumed to be constant [24]. Here, we aim to test the above assumptions (summarized in Table 1) against a large variety of data. The premise is that identification of assumptions supported by the data will facilitate the development of a single measurement model that can be reliably used for both fitting and model selection across a wide range of lymphocyte studies.

**Table 1:** Assumptions involved in previous measurement models (used either for model fitting or model selection).

Approach	Assumptions	Ref.
sum of squared residuals (SSR)	multivariate normal (MVN) distribution of measurements; diagonal covariance matrix; same marginal variances for all dimensions and time points	[11]
log-transformed sum of squared residuals (LogNrm)	multivariate lognormal distribution of measurements; diagonal covariance matrix for log-transformed data; same marginal variances for log-transformed samples	[10]
linear variance scaling (LVS)	MVN distribution of measurements; diagonal covariance matrix; linear relationship between marginal variances and means	[24]

Having introduced the prior art, we can now elaborate on the question that we address in this work. Given data and a set of predicted mean cell counts produced by a response model (e.g., Cyton or multitype branching process), we ask which objective function one should use for parameter estimation. Different previous models listed in Table 1 will lead to different objective functions (in this case, likelihoods). As we show in subsequent sections, the assumptions behind some of these likelihood functions are inconsistent with the data, and the choice of the function makes a difference to the final solution. Next, we present a new measurement model and the corresponding likelihood function. Finally, note that different likelihoods, including the likelihood derived from our model, can be used in situations where single or multiple parameter sets that maximize the likelihood are explored (search for global and local maxima, respectively).

## Results

We start with outlining the notation principles used in this paper. We use lowercase symbols to denote constant scalar values (e.g., *x*, *σ*) which can be vector or matrix components (e.g., *x*_*i*_, *σ*_*ij*_), symbols in boldface to denote constant vectors and matrices (e.g., *x*, *Σ*), uppercase symbols to denote random variables (e.g., *X*, Δ), and blackboard bold symbols for sets (e.g., D). For multivariate random variables, indexing can be used to denote a marginal variable (e.g., *X*_*i*_, *Δ*_*j*_). Furthermore, sample parameter estimates are denoted using a hat (e.g., $\hat{x},\hat{\mu }$). Finally, $R^{p}$ denotes *p*−dimensional Euclidean space, and $R^{p+}$ denotes a non-negative Euclidean subspace, i.e., for a *p*−dimensional vector, $x\in R^{p+}$ means that *x*_*i*_ ≥ 0, *i* = 1, …, *p*.

### Experimental data

A dataset is a set of samples $D=\{T_{\left(1\right)},\ldots ,T_{\left(d\right)}\}$, where each sample $T_{\left(t\right)}=\left\{z_{\left(t,1\right)},\ldots ,z_{\left(t,s_{t}\right)}\right\}$ is a set of *s*_*t*_ repeated measurements (replicates) taken at a distinct time point. We assume that all measurements from the same time point are generated by the same distribution that characterizes the lymphocyte response at that time point. Samples from different time points may come from different distributions. Each measurement ***z***_(*t*, *r*)_ is a *p*-dimensional vector, where component *z*_(*t*, *r*), *i*_ denotes the estimated number of cells that belong to group *i*. Examples of groups include cells that have divided two times since the beginning of the experiment, cells that have differentiated, or cells that have returned to quiescence. Different experiments can measure different quantities and result in datasets covering different groups. A range of fluorescent probes and semi-manual gating are used to assign cells to groups. Note that *z*_(*t*, *r*), *i*_ cannot be negative, but can be a fraction, because the estimation involves normalization using calibration bead counts [27].

Consider a lymphocyte response model with parameter vector ***θ*** (e.g., Cyton model with parameters such as mean time to first division, mean division destiny, etc. [18]; or a branching process with probabilities of division, death, etc. [7, 11, 13]). A probabilistic parameter estimation approach first aims to define the likelihood L(θ|D)≡Prob(D|θ). The experiments can be performed *in vitro* or *in vivo*. Here we focus on widely used protocols where repeated observations are measured from distinct physically separated wells *in vitro* or from different animals *in vivo* [4, 24]. Therefore, it is reasonable to assume independence, conditioned on a model with fixed parameter values, not only for different time points, but also for observations (i.e., replicates) within the same time point. More discussion on this assumption is provided in S2 Text. That is, we assume that (a) observations for different time points are independent: $Pr(T_{\left(i\right)},T_{\left(j\right)}|\theta )=Pr(T_{\left(i\right)}|\theta )Pr(T_{\left(j\right)}|\theta )$, *i* ≠ *j*, and (b) the repeated observations ***z***_(*t*, *r*)_ for a given time point *t* are independent and identically distributed (i.i.d.). Therefore, if *Z*_(*t*)_ denotes the measurement random variable (that incorporates both biological variation and experimental noise), we can write

$$L\left(\theta |D\right)=\prod_{t=1}^{d}\left(\prod_{r=1}^{s_{t}}P\left(Z_{\left(t\right)}=z_{\left(t,r\right)}|\theta \right)\right)$$ (1)

In order to explore the properties of distribution of *Z*_(*t*)_ in real world situations, we collect data from a number of independent experiments (either from published sources or executed for this study). We aim to collect data that represent a large variety of conditions encountered in lymphocyte studies (Table 2), and cover a range of cell types (wild type B or T cells, as well as knock outs), stimulation protocols, and treatments *in vitro* and *in vivo*. Importantly, the definition of a group (i.e., what is being measured) varies across experiments. For example, experiments *b–cd40* and *t–n4* represent typical proliferation assays based on the use of CFSE or CTV dyes. In these, and similar experiments, for each time point, *Z*_(*t*)_ is a vector comprising the estimated number of cells per generation. In contrast, measurements *Z*_(*t*)_ in dataset *t–vv–qsc* are two-dimensional vectors containing total cell number, as well as the number of cells returned to a quiescent state. Quiescent cells were estimated based on FUCCI expression and cell size. Six datasets were obtained from previous studies, while the remaining four experiments were conducted specifically for this study. These include a proliferation assay with 9 replicates per time point (dataset *b–bimko*). A typical number of replicates (*s*_*t*_) is 3 to 5, and a dataset with a large number of replicates is useful in studying the distribution of measurements. Finally, we include two experiments where proliferating B cells are treated with mitotic inhibitors (*b–gfp* and *b–tot*). These two datasets cover a combination of drugs, but contain only one time point for each condition. All data can be found in S1 Dataset.

**Table 2:** Summary of experimental data used in this work.

Dataset	Cells	Summary	Groups	Ref.
*b–cd40*	B cells	*In vitro* stimulation with anti-CD40 in the presence of interleukins 4 and 5 (4 conditions)	cell number per generation (CFSE)	[24]
*b–cpg*	B cells	*In vitro* stimulation with LPS (5 conditions)	cell number per generation (CFSE)	[24]
*b–lps*	B cells	*In vitro* stimulation with CpG (3 conditions)	cell number per generation (CFSE)	[24]
*t–il2*	OT-I Bim^-/-^ CD8+ T cells	*In vitro* stimulation using cognate peptide and various concentrations of interleukin 2 (8 conditions)	cell number per generation (CTV)	[4]
*t–vv–qsc*	OT-I FUCCI R/G CD8+ T cells	*In vivo* stimulation via HKx31-N4 influenza infection (2 conditions)	total cell number, quiescent cell number	[4]
*t–vv–tot*	IL2R*α*^+/+^ or IL2R*α*^-/-^ OT-I CD8+ T cells	*In vivo* stimulation via HKx31-N4 or HKx31-Q4 influenza infection (3 conditions)	total cell number	[4]
*b–bimko*	Bim^-/-^ and Bim^-/+^ B cells	*In vitro* stimulation with CpG (1 condition)	cell number per generation (CTV)	this work
*b–gfp*	Blimp-GFP B cells	B cells stimulated with anti-CD40 *in vitro* and treated with mitotic inhibitors Etoposide or Purvalanol A	total cell number, number of GFP+ cells	this work
*b–tot*	B cells	B cells stimulated with anti-CD40 *in vitro* and treated with mitotic inhibitors Aphidicolin, Etoposide, or Purvalanol A	total cell number	this work
*t–n4*	CD8+ T cells	*In vitro* stimulation with SIINFEKL peptide (1 condition)	cell number per generation (CTV)	this work

### Mean–variance relation

**Fig 3:**
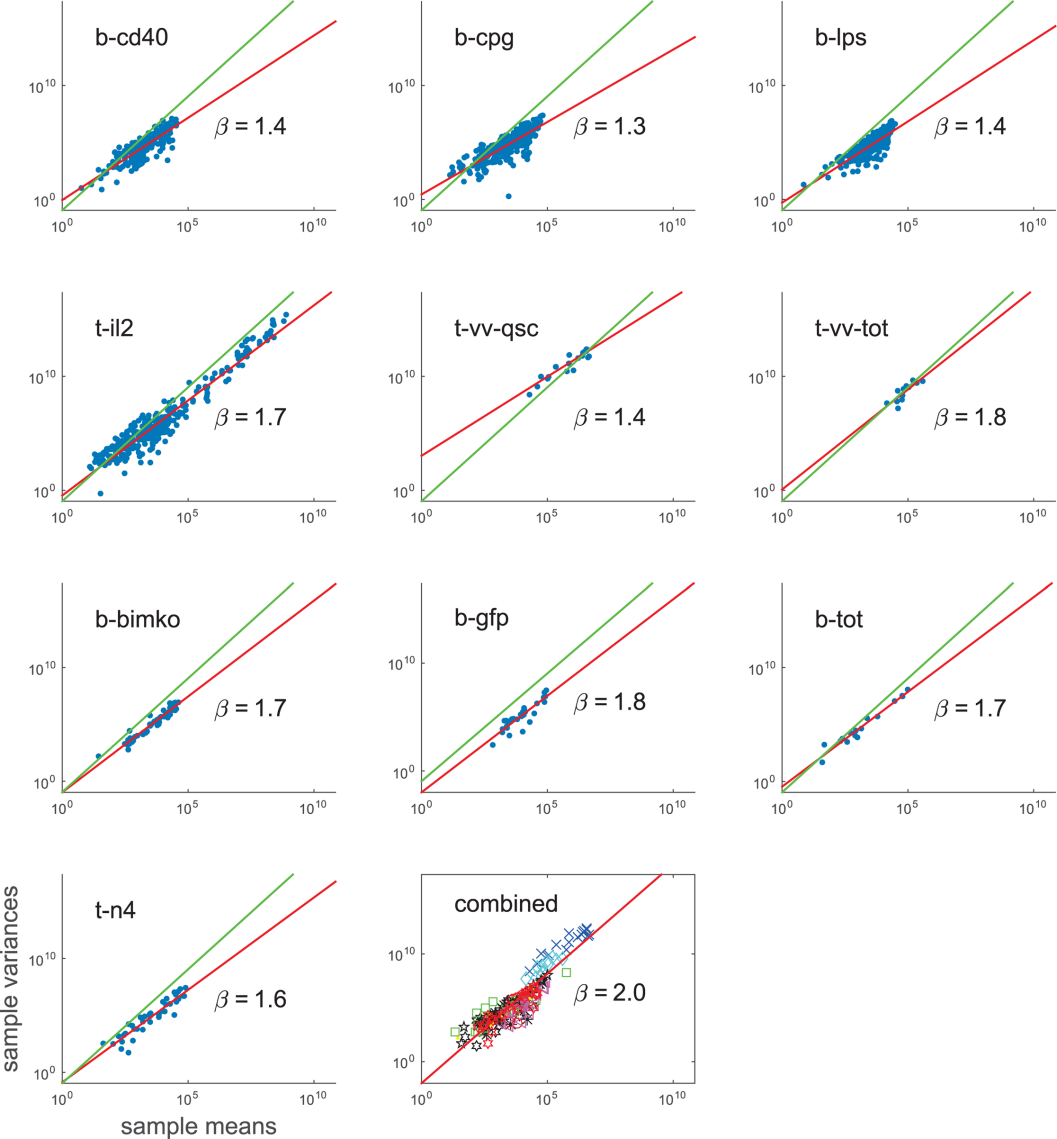
Empirical power-law relation between the mean and variance of measurements. Each point represents a single component of a sample of repeated measurements (replicates). For example, a point can represent repeated estimates of the number of cells falling in the GFP+ gate, or the number of cells in generation two for a particular time point. Each plot corresponds to all measured groups and time points for a particular dataset. The combined plot is composed of a collection of 20 random points sampled uniformly from each dataset. Red lines show fitted power law relations (*variance*) = *α*(*mean*)^*β*^, and the fitted power exponent is indicated for each dataset. Green lines show function (*variance*) = (*mean*)^2^/9. Most of the points are located below the green line, indicating that sample means tend to be larger than 3 standard deviations. If one assumes a Gaussian shape for the distribution of the measurements, this tendency indicates that most of the distribution mass is located in the positive Euclidean subspace.

Previous approaches have made specific assumptions about the marginal variances of *Z*_(*t*)_ (Table 1). Therefore, we first inspect the relation between means and marginal variances for each vector component for samples $T_{\left(t\right)}=\left\{z_{\left(t,1\right)},\ldots ,z_{\left(t,s_{t}\right)}\right\}$. Our datasets represent a range of experimental conditions and measured quantities. Strikingly, despite this variety, measurements from all experiments tend to follow a power law (Fig 3 and S1 Table). Marginal variances of measurements, as well as log-transformed measurements span several orders of magnitude and there is a statistically significant correlation with measurement means (S1 Fig and S1 Table). As such, constant variance assumptions of SSR and LogNrm methods are strongly violated. Moreover, we estimate the power exponent using a linear regression on a log-transformed axes, and find that in almost all datasets 95% confidence intervals for the estimated power exponent do not include 1, which is not consistent with the linear scaling assumption of LVS model. We conclude that the data does not support any of the previously used assumption sets listed in Table 1.

Points in Fig 3 can represent different quantities, such as the number of cell in a particular generation, or the number of differentiated cells. It is therefore interesting that all experiments exhibit a similar pattern. For example, residuals after power law fits tend to follow the same distribution across experiments (Kruskal-Wallis test p-value 0.998, number of points ranging from 12 to 321); and in each experiment, residuals tend to be centered around 0 (sign test p-values above 0.02 for each dataset, see S1 Table). Consistent with a previous observation [22], this may indicate that the experimental error originating from sources common to all datasets dominates variation in measurements. Such sources can include pipetting error, sample recovery by the flow cytometer, and effects of manual gating. Regardless of whether variability in these data originated mostly from biology or experimental procedure, we make an empirical observation that superficially measurement variance scales with mean according to a power law (that can have a different exponent for different datasets).

### Application of the maximum entropy principle

Computing the likelihood of parameters requires specification of the shape of a multivariate distribution of measurements *Z*_(*t*)_, and an MVN with independent components is a popular choice [11, 24, 28]. The literature on computational immunology says little about the justification of these assumptions. The central limit theorem can be used to argue for an MVN-distributed *true* cell numbers, but this argument is difficult to apply for measurements that include various sources of experimental errors. Therefore, we find it useful to present a systematic approach for justifying the choice of measurement distribution in the context of lymphocyte responses.

In principle, for each experimental scenario, one could perform a large number of replicates to infer the distribution. However, this approach is not practical, due to a large number of possible scenarios (e.g., those listed in Table 2). Therefore, we start with a theoretical argument justifying a particular choice of the distribution *a priori*. Further, we have implemented one experiment with 9 replicates, and in three other datasets we have samples with 5 or 6 replicates (dataset *b–bimko* in Table 2), and we use these data to test the *a priori* hypothesis.

When the information is incomplete (we do not know the true distribution), but a decision has to be made (we need to compute the likelihood), the safest choice would be to minimize the amount of additional information introduced by the decision. A formalization of this principle is the well known principle of maximum entropy [29]. Suppose we have a method for predicting mean and covariance of *Z*_(*t*)_ as a function of model parameters. Since measurements are non-negative quantities, among all possible probability distributions with the given first and second moment, a truncated multivariate normal (TMVN) distribution has the maximum differential entropy (S2 Text). Thus, according to the principle of maximum entropy, TMVN is a feasible choice for the distribution of measurements *a priori*. Interestingly, our data suggests that if measurements follow a TMVN distribution, most of the mass of the underlying MVN is located in $R^{p+}$ (in Fig 3, means tend to be larger than 3 standard deviations).

Therefore, we adopt multivariate normality as a reasonable approximation for measurements. In most practical cases, the number of replicates will not be sufficient for reliable selection of the distribution based on data, and this is where the principle of maximum entropy can be useful. In experiments with large numbers of replicates the choice of the distribution can be guided by data. In our data collection, we have only several time points (from different experiments) with more than 5 replicates, and we have assessed normality on these data (S3 Text). In most cases, the data was consistent with the proposed MVN distribution, and we therefore decided to use it in all cases for consistency. Future measurement models may depart from this decision by using, for example, mixture models, or selecting the shape of the distribution separately for each time point.

The principle of maximum entropy is also implemented in Dempster’s covariance selection method [30]. Reliable estimation of off-diagonal covariance elements of *Z*_(*t*)_ is challenging, given that in typical experiments the number of replicates is smaller than the number of dimensions. We observe that in the situation where marginal variances can be predicted, and off diagonal elements are unknown, Dempster’s method suggests a diagonal covariance matrix as the most feasible choice (S2 Text). We conclude that the principle of the maximum entropy provides a systematic way for justifying the shape of the distribution of measurements.

### Allowing non-zero means for experimental error

To summarize, given a dataset D, a ML or Bayesian parameter estimation routine can use the likelihood estimated as follows: (1) for each time point, compute ***η***_*t*_(***θ***) = *E*[*Z*_(*t*)_|***θ***], where ***θ*** is the candidate parameter vector; (2) estimate dataset-specific power law parameters *α* and *β* from D; (3) estimate marginal variances *υ*_*ii*_ = *α* ⋅ *η*_*t*_(***θ***)^*β*^; and (4) compute likelihood L(θ|D) using Eq 1 and using the density of MVN with a diagonal covariance matrix.

**Fig 4:**
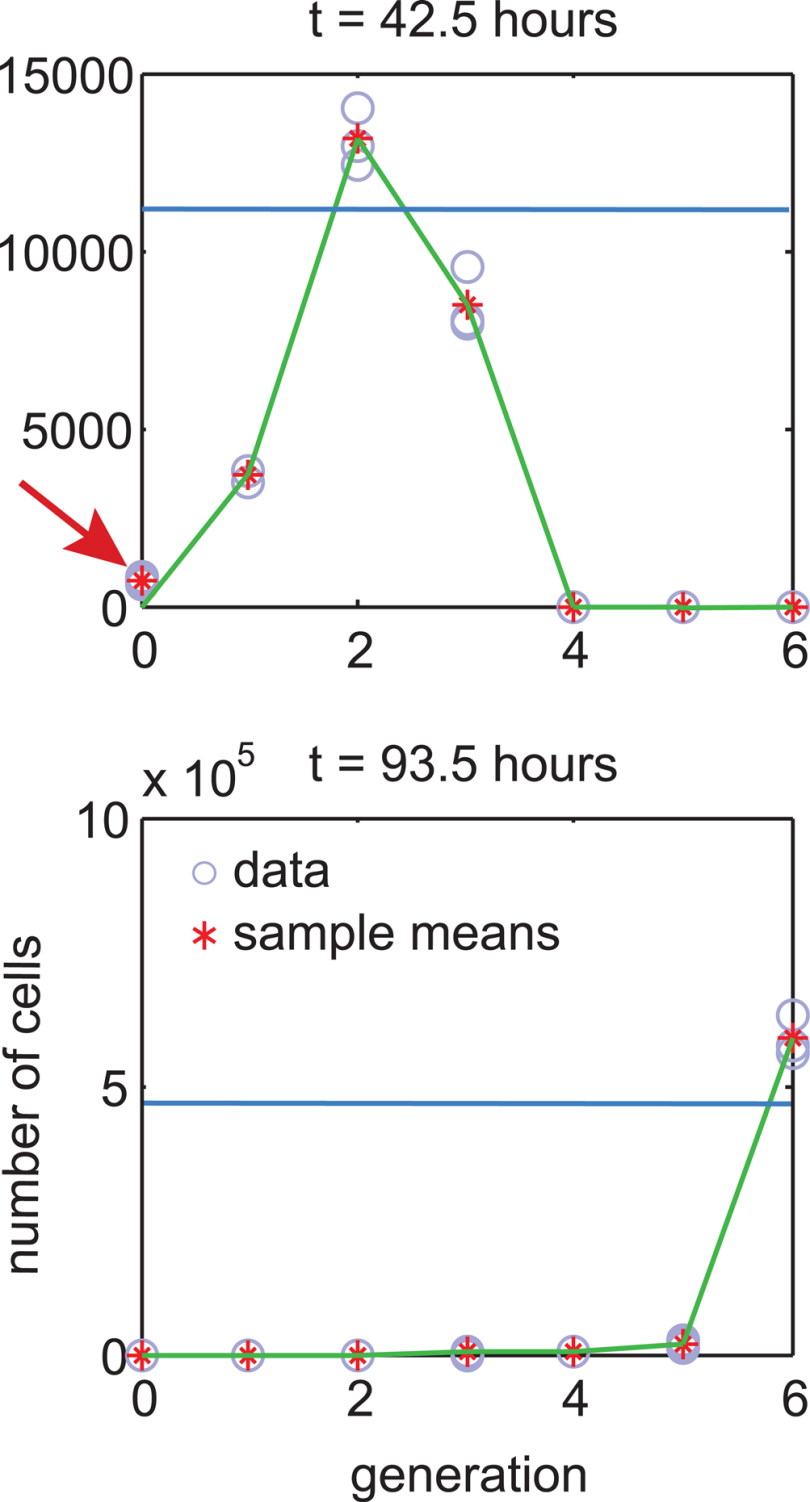
The problem of assuming zero mean for experimental error. Consider two fits to the same data extracted from *t–il2* dataset for 316 U/mL condition. In this experiment, a data sample comprises repeated estimates of number of cells in a particular generation. The green fit goes through all generation means, except for the first sample pointed by the arrow. The blue fit predicts an arbitrary value everywhere. Intuitively, the green fit is more feasible, but the blue fit is more likely. This happens because for the first sample variance is close to zero which makes the whole green fit nearly impossible. In practice, mismatch at the first sample can be attributed to a non-zero mean of modeling or experimental noise.

Going back to step one, consider a lymphocyte response model that predicts means ***μ***_*t*_(***θ***), and assume that prediction is perfect, and the experimental error has a zero mean, i.e., ***μ***_*t*_(***θ***) = *E*[*Z*_(*t*)_|***θ***]. This assumption can lead to an anomaly illustrated in Fig 4. In the figure, the predicted mean is small and so is the predicted variance. Therefore, the likelihood of the green fit is essentially zero, despite the fit visually appearing to be good.

A possible solution to this problem is to assume a minimum measurement variance, i.e., *υ*_*ii*_ = *max*(*c*, *α* ⋅ ***η***_*t*_(***θ***)^*β*^) [24]. However, this approach contradicts the data. For example, dataset *t–il2* alone has over 10 samples consisting of all zeros (i.e., {0, 0, 0}). All-zero samples are also observed in other datasets, which suggests that when the real number of cells is close to 0, so is the measurement variance. Instead, we note that the real reason, the green fit in Fig 4 can be considered feasible, is understanding that (a) a response model is never a perfect representation of reality, and (b) the mean of experimental error can be non-zero. In other words, there can be a difference between the mean of the measurement distribution, and the predicted mean of the response model. We call this difference *measurement offset*, and propose to model measurement means as ***η***_*t*_(***θ***) = ***μ***_*t*_(***θ***)+***δ***, where ***δ*** represents the offset. This offset can be estimated from the data, but needs specification of a prior distribution (otherwise an arbitrary fit can be considered a good fit with a large offset). We developed a maximum *a posteriori* (MAP) parameter estimation method, where user needs to specify a reasonable variance on *δ* (S2 Text). This is the only user-provided input (denoted *ε*) in the our parameter estimation routine, and the choice of *ε* is guided by data. We call our measurement model a three factor (3F) model, because it accounts for the lymphocyte response model, modeling error, and experimental error (S2 Text).

### Evaluation of measurement models

Our ultimate aim is to fit a response model to experimental data. To this end, we take a probabilistic approach where a response model is embedded within a measurement model (Fig 2), and focus on different choices for the measurement model. In this section, we assess the performance of the 3F model and its applicability to fitting. Note that in principle, our conclusions are not restricted to any particular *response* model. For this evaluation, two previous models were chosen as representative examples, namely Age Dependent Multitype Branching Process (ADMBP) as summarized in reference [11], and the Cyton model as defined in reference [4]. Both models predict the number of cells per generation over time. The Cyton model with 12 parameters is more elaborated than the ADMBP model with 11 parameters. However, note that here we do not aim to compare proliferation models.

In regards to measurement models, we consider those listed in Table 1. For the purposes of evaluation, it is tempting to generate synthetic data with known response parameters, and assess how these parameters are recovered by different measurement models. However, it is not clear how to generate realistic measurement noise on synthetic data in the first place, since using any of known measurement models, would make the evaluation in favor of that model. Instead, we assess model performance using AICc, and also discuss some theoretical aspects of fitting using different measurement models. In addition, we present a quantitative evaluation using a hypothetical ideal response model. For a given dataset, the hypothetical model simply yields predictions equal to sample means from that dataset. In other words, the hypothetical model mimics the behavior of some accurate model, because an accurate model would be expected to predict values close to the sample means. By the way it is defined it will always outperform any actual model, such as Cyton or ADMBP in terms of the likelihood of resulting fits. In terms of the AICc, the “number of parameters” for the hypothetical model is controlled artificially.

**Fig 5:**
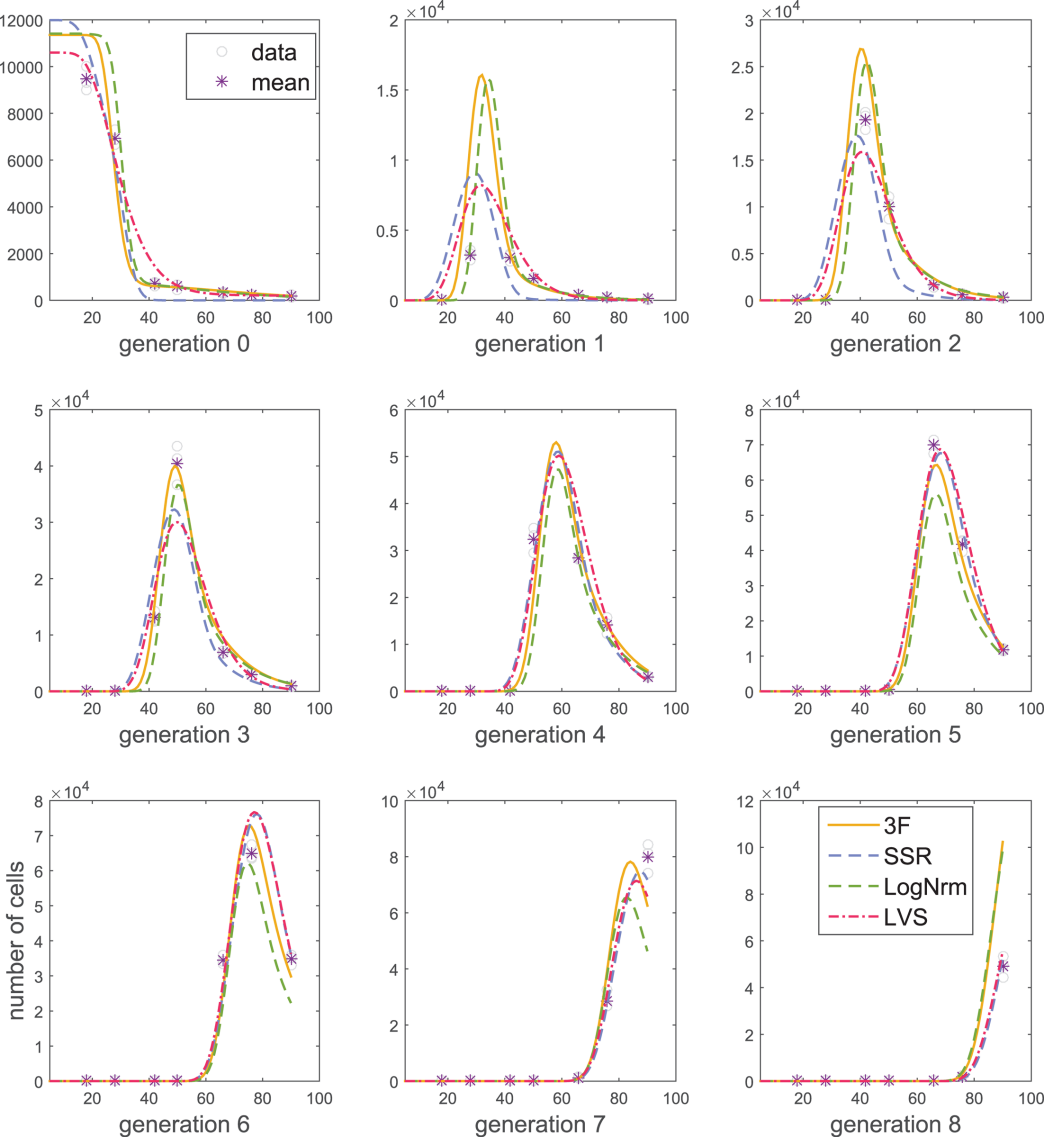
Model fitting using different measurement models. The Cyton model was fitted to *t–n4* data using different measurement models. Resulting AICc scores are 2050.7 (3F), 3633.8 (SSR), 4001.7 (LogNrm), and 109231.8 (LVS). For the fit obtained using LogNrm model, AICc was computed using the SSR measurement model.

Best fits obtained using all four measurement models for representative datasets are shown in Fig 5 and S2–S4 Figs. In each figure, the data and response model are fixed, and the only difference between the fitted lines is the assumptions about measurement noise. Note that the choice of the measurement model can lead to different fits (see also multiconcentration example below). Further, note that visually use of the 3F model results in good fits compared with other measurement models (the chosen response models do not necessarily allow perfect fits). Quantitatively, the 3F measurement model offers the best explanation for the data as suggested by AICc scores.

Next, we note two theoretical inconveniences of the LogNrm model. First, this model assumes that response predictions, such as those produced by ADMBP or Cyton models are medians of measurements, whereas most proliferation models predict the means of the measurements [4, 11]. A more severe inconvenience stems from the need of data capping for log-transformation (S1 Text). Information criteria, such as AICc and BIC cannot be used to compare fits performed on capped and original data, or data capped at different levels. Therefore, in this section, for fits obtained using LogNrm model, we report the AICc computed using a SSR model.

**Fig 6:**
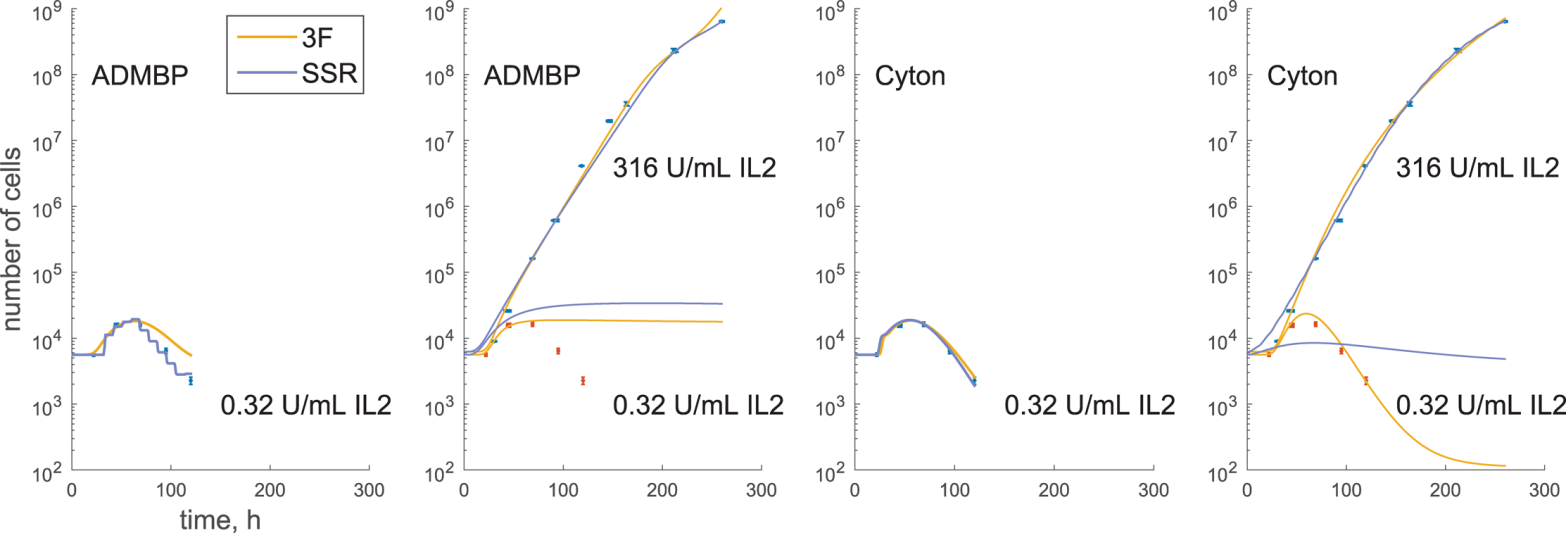
SSR is not suitable for multiconcentration fitting. ADMBP and Cyton models are fitted to a low and high stimulation condition selected from *t–il2* dataset. When a low concentration data is fitted independently, SSR can be used to fit models. However, when both concentrations are fitted simultaneously, SSR tends to ignore low concentration data because the range of these data is comparable to measurement noise for the high concentration measurements. At the same time, 3F tends to produce more balanced fits, because it scales measurement variance in accordance to the mean.

Further, for measurement means close to zero (e.g., 0.001), LVS and SSR models allow positive variances (e.g., 10), thus predicting an approximately 50% probability of a negative measurement. This formulation contradicts the data, and can be one of the reasons for reduced likelihood, compared to the 3F model (Fig 5). Moreover, fits using SSR treat all measurements as equally important (having equal variance), which can lead to undesirable situations where a model cannot predict values close to measurement means for all time points simultaneously. This can be a confounding factor especially for multiconcentration fitting. A multiconcentration fitting is an emerging problem [4, 24], where an experiment is repeated with one of the variables changed, e.g., T cells proliferate in the presence of different concentrations of interleukin 2 (IL2). A response model can be then fit simultaneously to the data for all concentrations, where certain proliferation parameters (e.g., starting cell number) are constrained to remain the same across concentrations. In addition, in a multiconcentration experiment, it is common to observe large differences in cell numbers, where weak stimulation results in rapid population extinction, whereas string stimulation leads to a considerable growth. The use of SSR for such data may result in the low concentration data being essentially ignored (Fig 6).

So far, we have considered ADMBP and Cyton response models. Note that for a given dataset, an ideal response model would be the one that predicts measurement means. We therefore, also implemented such a hypothetical model, and assumed that it has *k*_*resp*_ parameters, where *k*_*resp*_ was set to 1%, 5% or 10% from the number of data points. We then embedded this model within 3F, SSR, or LVS, and computed AICc for each dataset. We find that overall 3F offers the best explanation for the data (S2 Table).

Finally, note that embedding a response model within a measurement model may increase the total number of parameters. Additional parameters introduced by the 3F model are discussed in S2 Text. All of them, except one (denoted *ε*) are estimated from the data. In our evaluation, we use the default value ε* = 50. Further, in S2 Text we discuss situations when fitting is not expected to be sensitive to the choice of *ε*. Indeed, when we repeated fitting several times, each time starting from the same initial parameter guess, but varying *ε* from 0.25ε* to 4ε*, we obtained similar results (S5 Fig). Overall, we conclude that the 3F measurement model offers a reliable probabilistic way of fitting response models to lymphocyte data. This measurement model tends to explain the data better than previous models, and does not suffer certain undesirable effects, such as data capping or imbalanced fitting between data for different stimuli concentrations.

## Discussion

Flow cytometry is a popular tool for quantifying lymphocyte responses—and fitting mathematical models to these data is a common practical problem. Experimental noise originating from pipetting, recovery and multiple gating errors poses a major challenge for fitting models to such data. A common way to overcome this difficulty is to make simplifying assumptions as to the distribution of measurements. The lack of a systematic assessment of variability in measurements from lymphocyte response experiments has led to the situation where different research groups have adopted different sets of assumptions for fitting to the same type of data. A further inconsistency with these methods is that fitting is sometimes performed using a heuristic objective function, while the resulting fits are compared using a probabilistic criterion.

Here we addressed this problem by assessing commonly used assumptions on measurements. We collected data representative of a wide range of experimental conditions and found an empirical power law relation *υ* = *αμ*^*β*^, where *μ* is the sample mean and *υ* is the sample variance for measured replicates from each group (e.g., a sample of repeated measurements for cells in generation 2 at 24 hours). From our data, we estimated *β* to fall between 1.3 and 1.8 (it varies for different datasets) which violates common assumptions of the variance being constant (SSR) or scaling linearly with the mean (LVS), as well as the assumption of log-normal measurements (LogNrm).

Mathematically, the observed mean-variance relation is equivalent to the well-known empirical Taylor’s law that has been found to apply in various biological domains [31–33]. Taylor described this law in an ecological context, counting the number of species per unit area, and related the power exponent to the degree of aggregation of the organisms. The emergence of a power law in the context of lymphocyte experiments is presumably due to completely different reasons that are yet to be established. It is also likely that these reasons primarily reflect idiosyncrasies of flow cytometry/gating workflow rather than biology, in which case, the main interest in the power-law relation is for model fitting. Indeed, in our measurement model we estimate *α* and *β* from data which enables us to achieve more reliable model fitting compared to methods where *α* and *β* are fixed.

Multiconcentration fitting is an emerging task where a range of conditions, such as titration of a cytokine, is tested within a single experiment. A fit is then performed simultaneously to the results of all conditions, with some parameters (e.g., death rate) constrained to remain constant across conditions. Since cell counts are likely to differ by several orders of magnitude for different concentrations it is important to balance the contribution of each data point. Allowing power law scaling helps to achieve this balance in our measurement model.

In our measurement model, the power law exponent is estimated independently for each dataset, but is fixed for a dataset. Furthermore, we assume a single-component MVN distribution for all measurements. This decision was made in view that most of lymphocyte response models assume a homogeneous population of cells with a fixed parameter vector describing the population [4, 7, 11]. Our evaluation suggests that even this simple setup can lead to improvements compared to the state-of-the-art. At the same time, future and more advanced measurement models can consider, for example, specifying a distribution over the power law exponent, or a mixture model for measurements as a consequence of presence of different cell types within a population. Another possibility to explore is letting the power exponent to vary over time (S6 Fig). Our current work contributes an evaluation framework, and a new baseline facilitating progress in the field.

In addition to the applications detailed above, we have addressed the theoretical foundation of our method. We justify the choice of the measurement distribution using the maximum entropy principle. This is a well-known principle in the field of signal processing, and here we introduce this idea in the context of quantifying lymphocyte responses. Further, the theoretical development of our model is not specific to lymphocytes and potentially can be applied to flow cytometry experiments involving other cell types. In regards to lymphocyte responses, our work lays a foundation for a consistent handling of flow cytometry data to support ongoing and future model development in the field.

## Materials and Methods

### Mice, culture preparation, flow cytometry

Our dataset collection comprises 6 datasets from previous studies, and 4 datasets performed for this study, and outlined below. All experiments were performed under the approval of the Walter and Eliza Hall Institute (WEHI) Animal Ethics Committee. All mice used were maintained in specific pathogen-free conditions at WEHI animal facilities (Kew, Bundoora, and Parkville, Victoria, Australia) in accordance with WEHI animal ethics committee regulations. All transgenic mouse strains used were on a C57BL/6 background.

#### b–bimko

Small resting B cells, isolated from spleens from one B6/del339-/- (C57BL/6, deficient for Bim) and one B6/del339+/- (heterozygous for Bim deficiency) were labelled with 5μM CTV and stimulated with 3μM CpG 1668 at 10,000 cells per well. Cell isolation procedure and culture conditions were as described previously [21]. Measurements were performed in 9 replicates.

#### *b–gfp* and *b–tot*

Small, resting naÃ¯ve B cells were isolated from the spleens of Blimp-1^*GFP*/+^ reporter mice at 10–12 weeks of age using a percoll gradient (65/80% interface) and magnetic-activated cell sorting B cell isolation kit (Miltenyi). Cells were stained with 7.5 *μM* CTV and placed into culture with 10 *μg*/*mL* anti-CD40 antibody (1C10) and 1000 U/mL IL-4. Cells were bulk cultured at 100,000 cells/mL for 3 days (under 37°C, 5% *CO*_2_, and humidity control) before being harvested and plated at 20,000 cells per 200 *μL* in triplicate wells, in the presence of stimuli as before, as well as cell-cycle inhibiting drugs at various concentrations (*b–gfp*: etoposide, purvalanol A and vincristine; *b–tot*: etoposide, purvalanol A and aphidicolin). Further, 6,000 fluorescent beads were added to each well in 10 *μL* just prior to analysis at day 4 (*b–gfp*) or day 7 (*b–tot*) on the BD Canto flow cytometer, along with 0.5 *μM* of propidium iodide.

#### t–n4

Naive CD8 T cells were isolated from the lymph nodes of approximately 12 weeks old OT-1 mice using a MACS CD8 II isolation kit. Anti-CD44-biotin antibody was added to biotinylated purification cocktail to remove memory T cells. Purified naive CD8 T cells were labelled with 5μM CTV and cultured at 10,000 cells /well in the presence of 1ng/mL IL-7 and stimulated with 10ng/mL SIINFEKL peptide and 100U/mL IL-2. Cells were incubated in a humidified atmosphere at 37°C and 5% CO2. At the time points indicated 10,000 beads and 0.2% propidium iodide were added to each well immediately prior to analysis on a BD Canto flowcytometer. Calculation of total cell numbers and cell numbers per division are described in [4].

Datasets *b–cd40*, *b–cpg*, and *b–lps* were obtained from the authors of [24]. These data are shown, respectively, in Supplementary Figures S4, S2 and S3 in reference [24]. Datasets *t–il2*, *t–vv–qsc*, and *t–vv–tot* were obtained from the authors of [4]. These data are shown, respectively, in Figures 2C, 1D and 4D in reference [4].

### Statistical analysis of the datasets

For the statistics presented in S1 Table, Pearson correlation p-values were computed using a Student’s t distribution for a transformation of the correlation. Power law parameters were estimated using linear regression with two parameters (slope and intercept) on the log-log scale. This estimation was performed separately for each dataset. Confidence intervals for linear regression were estimated as *b*_*i*_±*t*_(1−*α*/2, *n*−*c*)_ *SE*(*b*_*i*_), where *SE*(*b*_*i*_) is the standard error of the estimate, *t*_(1−*α*/2, *n*−*c*)_ is the 100 × (1−*α*/2) percentile of *t* distribution with *n*−*c* degrees of freedom, *n* is the number of observations, and *c* is the number of regression coefficients.

### Parameter estimation

Parameter estimation (model fitting) was performed as an optimization that maximizes the likelihood (for SSR, LogNrm, and LVS models) or maximum *a posteriori* probability (for 3F) as a function of *θ*. The optimization was implemented and tested in a MATLAB 2014b environment using the implementation of the interior point algorithm provided by the environment (via standard *fmincon* function). The optimization result depends on the starting point *θ*_0_ ∈ Θ [28], and we repeated optimization independently 10 times with randomly generated starting vectors. Random starting vectors have each of their components sampled independently from other components uniformly from the range of valid parameter values provided by the user. For multiconcentration fitting, some parameters can be *fixed*, i.e., constrained to remain equal across concentrations. These user-defined parameter ranges, as well as lists of fixed parameters can be found in S1 Dataset. Each of the runs returned an estimate for the optimal parameter vector, and the final result was the best vector across all runs. Source code for the fitting process can be found in S1 File. Note that LVS and LogNrm involve additional parameters, and we used *ε* = 50 for LVS, and $c=\sqrt{50}$ for LogNrm.

## Supporting Information

S1 Dataset Experimental data used in this work. Detail of data format can be found within the archive.
(ZIP)

S1 Fig Variance of log-transformed data is not constant. Plots show means and variances of log-transformed data for each of the datasets. Variance can span several orders of magnitude and it depends on the mean. This violates assumptions of LogNrm approach.
(PDF)

S2 Fig Model fits obtained using different measurement models. ADMBP model was fitted to *t–n4* data using different measurement models. Resulting AICc scores are 2320.7 (3F), 4046.2 (SSR), 4166.8 (LogNrm), and 311694.4 (LVS). For the fit obtained using LogNrm model, AICc was computed using SSR model.
(PDF)

S3 Fig Model fits obtained using different measurement models. Cyton model was fitted to *b–bimko* data using different measurement models. Resulting AICc scores are 8238.4 (3F), 10326.0 (SSR), 11602.7 (LogNrm), and 436803.5 (LVS). For the fit obtained using LogNrm model, AICc was computed using SSR model.
(PDF)

S4 Fig Model fits obtained using different measurement models. ADMBP model was fitted to *b–bimko* data using different measurement models. Resulting AICc scores are 9085.5 (3F), 12024.4 (SSR), 13125.6 (LogNrm), and 2674181.0 (LVS). For the fit obtained using LogNrm model, AICc was computed using SSR model.
(PDF)

S5 Fig Fitting is not sensitive to the choice of parameter *ε*. Cyton model was fitted to a representative dataset (in this case, *b–lps*), and the fitting was repeated several times using different *ε* ranging from 0.25ε* to 4ε*, where ε* = 50. The choice of *ε* made little difference to results. Note that this is a multiconcentration dataset, and the fit was performed to all concentrations simultaneously. Further, each time point contains breakdown of the number of cells per generation, but in the figure, only the total number are shown.
(PDF)

S6 Fig Analysis of the power law exponent over time. Each point shows the power law exponent estimated from samples from a particular time point. Any given time point comprises several measurement samples (e.g., number of cells in division 1 measured 3 times is one sample; number of cells in division 2 measured 3 times is another sample, etc.). Each sample is then summarized by sample mean and sample variance, a power law relation between mean and variance is fit to data from a single time point, and the exponent is plotted against time. We used only time points with more than 3 samples. Datasets *b-gfp* and *b-tot* did not have time points that satisfy this criterion. Overall, there appears to be a slight increasing trend for each dataset, and this observation can be exploited in future measurement models.
(PDF)

S1 File Source code for parameter estimation. MATLAB implementation of 3F measurement model, Cyton response model, as well as fitting and data analysis routines.
(ZIP)

S2 File Compiled executables. These files can be used to reproduce figures and tables from this paper, as well as for fitting to new data. Please refer to *readme.txt* for details.
(ZIP)

S1 Table Statistics computed for experimental data. For each dataset, power law was fitted as a linear regression on the log-log scale, and table shows the parameters of the regression. In the fitted line, slope corresponds to the power exponent and intercept corresponds to the logarithm of the scaling coefficient. Next, table shows p-values for the sign test for residuals of the linear regression, and p-values of Pearson correlation between sample means and variances. The final column shows p-values for Pearson correlation between sample means and variances for log-transformed data. The last row shows statistics for the combined dataset, and it contains p-value for Kruskal-Wallis test.
(XLSX)

S2 Table Comparing performance of measurement models. For each dataset, we consider the fit matching all measurement means, produced by a hypothetical response model with *k*_*resp*_ parameters. The number of response model parameters was set to 1%, 5% or 10% of the number of data points. Measurement model performance was assessed using AICc. Overall, 3F outperforms other models.
(XLSX)

S1 Text Previous measurement models. Formal description of some previously proposed measurement models.
(PDF)

S2 Text Description of our measurement model. Complete formal description of the proposed measurement model and parameter estimation methods.
(PDF)

S3 Text Testing normality of the data. Normality tests suggest that a MVN distribution is a reasonable approximation for the distribution of measurements. Unless otherwise stated, p-values are given for Lilliefors test.
(PDF)

## References

[1] Duffy K, Wellard C, Markham J. Activation-induced B cell fates are selected by intracellular stochastic competition. Science. 2012;335(6066):338–341. 22223740 10.1126/science.1213230

[2] Inokuma MS, Maino VC, Bagwell CB. Probability state modeling of memory CD8(+) T-cell differentiation. Journal of immunological methods. 2013 11;397(1–2):8–17. 10.1016/j.jim.2013.08.003 23954473

[3] Sciammas R, Li Y, Warmflash A, Song Y, Dinner AR, Singh H. An incoherent regulatory network architecture that orchestrates B cell diversification in response to antigen signaling. Molecular systems biology. 2011 5;7(495):495 Available from: http://www.pubmedcentral.nih.gov/articlerender.fcgi?artid=3130558&tool=pmcentrez&rendertype=abstract. 10.1038/msb.2011.25 21613984 PMC3130558

[4] Marchingo JM, Kan A, Sutherland RM, Duffy KR, Wellard CJ, Belz GT, et al Antigen affinity, costimulation, and cytokine inputs sum linearly to amplify T cell expansion. Science. 2014 11;346(6213):1123–1127. 10.1126/science.1260044 25430770

[5] Couzin-Frankel J. Cancer Immunotherapy. Science. 2013;342(6165):1432–1433. 10.1126/science.342.6165.1432 24357284

[6] Perfetto SP, Chattopadhyay PK, Roederer M. Seventeen-colour flow cytometry: unravelling the immune system. Nature reviews Immunology. 2004;4(8):648–655. 10.1038/nri1416 15286731

[7] De Boer RJ, Perelson AS. Quantifying T lymphocyte turnover. Journal of theoretical biology. 2013 1;. 10.1016/j.jtbi.2012.12.025 23313150 PMC3640348

[8] Hyrien O, Zand MS. A Mixture Model With Dependent Observations for the Analysis of CSFE–Labeling Experiments. Journal of the American Statistical Association. 2008 3;103(481):222–239. 10.1198/016214507000000194

[9] León K, Faro J, Carneiro J. A general mathematical framework to model generation structure in a population of asynchronously dividing cells. Journal of theoretical biology. 2004 8;229(4):455–76. 10.1016/j.jtbi.2004.04.011 15246784

[10] Luzyanina T, Cupovic J, Ludewig B, Bocharov G. Mathematical models for CFSE labelled lymphocyte dynamics: asymmetry and time-lag in division. Journal of mathematical biology. 2013 12; 24337680 10.1007/s00285-013-0741-z

[11] Miao H, Jin X, Perelson AS, Wu H. Evaluation of multitype mathematical models for CFSE-labeling experiment data. Bulletin of Mathematical Biology. 2012 2;74(2):300–326. Available from: http://www.pubmedcentral.nih.gov/articlerender.fcgi?artid=3196768&tool=pmcentrez&rendertype=abstract. 10.1007/s11538-011-9668-y 21681605 PMC3196768

[12] Shokhirev MN, Hoffmann A. FlowMax: A Computational Tool for Maximum Likelihood Deconvolution of CFSE Time Courses. PloS one. 2013 1;8(6):e67620 Available from: http://www.pubmedcentral.nih.gov/articlerender.fcgi?artid=3694893&tool=pmcentrez&rendertype=abstract. 10.1371/journal.pone.0067620 23826329 PMC3694893

[13] Yates A, Chan C, Strid J, Moon S, Callard R, George AJT, et al Reconstruction of cell population dynamics using CFSE. BMC bioinformatics. 2007;8:196 10.1186/1471-2105-8-196 17565685 PMC1929124

[14] Lyons aB, Parish CR. Determination of lymphocyte division by flow cytometry. Journal of immunological methods. 1994;171(94):131–137. 10.1016/0022-1759(94)90236-4 8176234

[15] Quah BJC, Parish CR. New and improved methods for measuring lymphocyte proliferation in vitro and in vivo using CFSE-like fluorescent dyes. Journal of Immunological Methods. 2012;379(1–2):1–14. 10.1016/j.jim.2012.02.012 22370428

[16] Revy P, Sospedra M, Barbour B, Trautmann a. Functional antigen-independent synapses formed between T cells and dendritic cells. Nature immunology. 2001;2(10):925–931. 10.1038/ni713 11561183

[17] Hyrien O, Mayer-Pröschel M, Noble M, Yakovlev A. A stochastic model to analyze clonal data on multi-type cell populations. Biometrics. 2005;61(1):199–207. 10.1111/j.0006-341X.2005.031210.x 15737094

[18] Hawkins ED, Turner ML, Dowling MR, van Gend C, Hodgkin PD. A model of immune regulation as a consequence of randomized lymphocyte division and death times. Proceedings of the National Academy of Sciences of the United States of America. 2007 3;104(12):5032–7. Available from: http://www.pubmedcentral.nih.gov/articlerender.fcgi?artid=1821128&tool=pmcentrez&rendertype=abstract. 10.1073/pnas.0700026104 17360353 PMC1821128

[19] Kallies A, Hasbold J, Tarlinton DM, Dietrich W, Corcoran LM, Hodgkin PD, et al Plasma cell ontogeny defined by quantitative changes in blimp-1 expression. The Journal of experimental medicine. 2004 10;200(8):967–77. Available from: http://www.pubmedcentral.nih.gov/articlerender.fcgi?artid=2211847&tool=pmcentrez&rendertype=abstract. 10.1084/jem.20040973 15492122 PMC2211847

[20] Sakaue-Sawano A, Kurokawa H, Morimura T, Hanyu A, Hama H, Osawa H, et al Visualizing spatiotemporal dynamics of multicellular cell-cycle progression. Cell. 2008;132(3):487–498. 10.1016/j.cell.2007.12.033 18267078

[21] Hawkins ED, Markham JF, McGuinness LP, Hodgkin PD. A single-cell pedigree analysis of alternative stochastic lymphocyte fates. Proceedings of the National Academy of Sciences of the United States of America. 2009;106(32):13457–13462. 10.1073/pnas.0905629106 19633185 PMC2715326

[22] Subramanian VG, Duffy KR, Turner ML, Hodgkin PD. Determining the expected variability of immune responses using the cyton model. Journal of Mathematical Biology. 2008 6;56(6):861–892. Available from: http://www.ncbi.nlm.nih.gov/pubmed/17982747http://www.springerlink.com/index/419W462T31U11661.pdf. 10.1007/s00285-007-0142-2 17982747

[23] Hasbold J, Gett aV, Rush JS, Deenick E, Avery D, Jun J, et al Quantitative analysis of lymphocyte differentiation and proliferation in vitro using carboxyfluorescein diacetate succinimidyl ester. Immunology and Cell Biology. 1999;77(6):516–522. 10.1046/j.1440-1711.1999.00874.x 10571672

[24] Hawkins ED, Turner ML, Wellard CJ, Zhou JHS, Dowling MR, Hodgkin PD. Quantal and graded stimulation of B lymphocytes as alternative strategies for regulating adaptive immune responses. Nature communications. 2013 9;4:2406 10.1038/ncomms3406 24009041 PMC3778729

[25] Banks HT, Kapraun DF, Thompson WC, Peligero C, Argilaguet J, Meyerhans A. A novel statistical analysis and interpretation of flow cytometry data. Journal of biological dynamics. 2013 1;7(1):96–132. Available from: http://www.pubmedcentral.nih.gov/articlerender.fcgi?artid=3753657&tool=pmcentrez&rendertype=abstract. 10.1080/17513758.2013.812753 23826744 PMC3753657

[26] Burnham KP, Anderson DR. Model selection and multi-model inference: a practical information-theoretic approach. 2nd ed Springer-Verlag; 2002.

[27] Hawkins ED, Hommel M, Turner ML, Battye FL, Markham JF, Hodgkin PD. Measuring lymphocyte proliferation, survival and differentiation using CFSE time-series data. Nature protocols. 2007 1;2(9):2057–67. 10.1038/nprot.2007.297 17853861

[28] Baker C, Bocharov G. Computational modelling with functional differential equations: Identification, selection, and sensitivity. Applied Numerical.… 2005;Available from: http://www.sciencedirect.com/science/article/pii/S0168927404001308.

[29] Jaynes ET. Information Theroy and Statistical Mechanics. The Physical Review. 1957;106(4):620–630. 10.1103/PhysRev.106.620

[30] Dempster A. Covariance selection. Biometrics. 1972;Available from: http://www.jstor.org/stable/2528966.

[31] Taylor LR. Aggregation, Variance and the Mean. Nature. 1961;189(4766):732–735. 10.1038/189732a0

[32] Kendal WS. A stochastic model for the self-similar heterogeneity of regional organ blood flow. Proceedings of the National Academy of Sciences of the United States of America. 2001;98(3):837–841. 10.1073/pnas.98.3.837 11158557 PMC14670

[33] Kendal WS. A scale invariant clustering of genes on human chromosome 7. BMC evolutionary biology. 2004;4:3 10.1186/1471-2148-4-3 15040817 PMC373443

