# Supplementary figures and images for: Stochastic Measurement Models for Quantifying Lymphocyte Responses Using Flow Cytometry
Source: PLoS One. 2016 Jan 7;11(1):e0146227. doi: 10.1371/journal.pone.0146227 (PMC4704825; doi:10.1371/journal.pone.0146227)

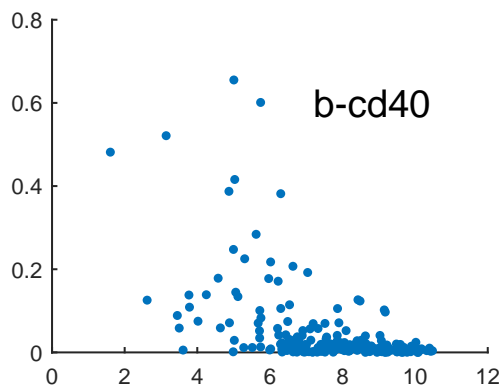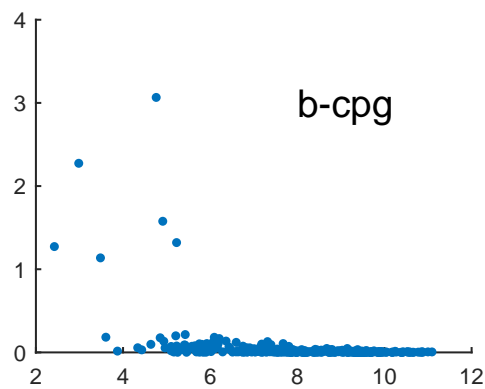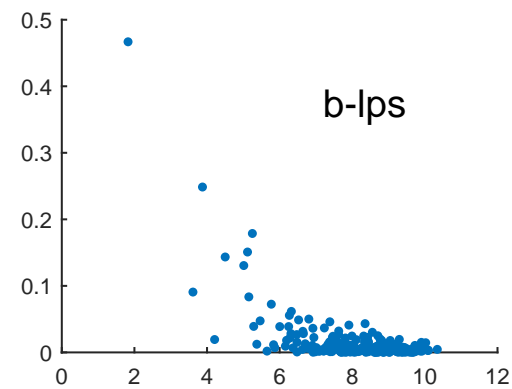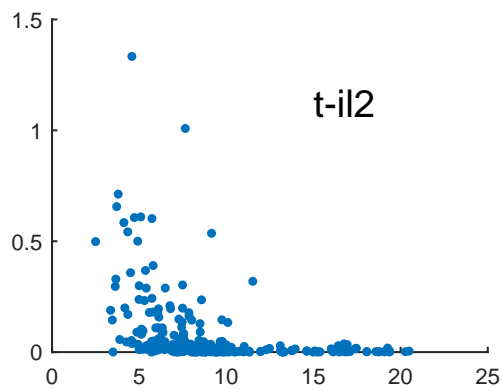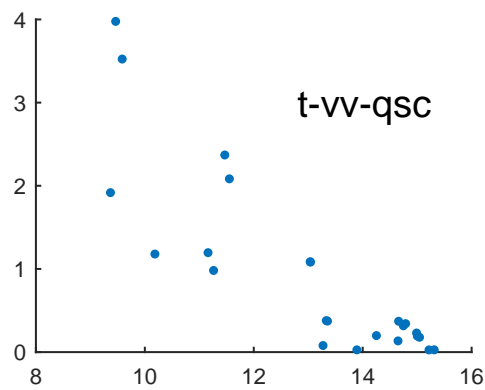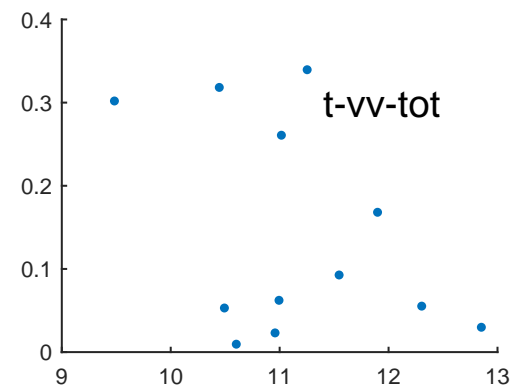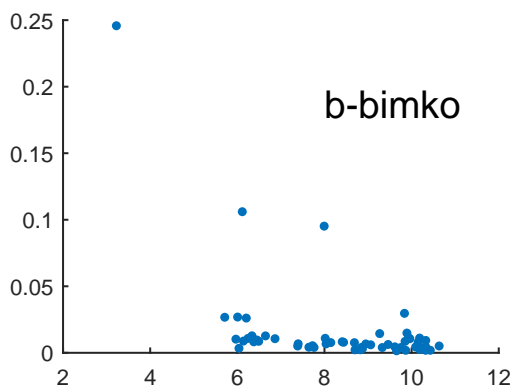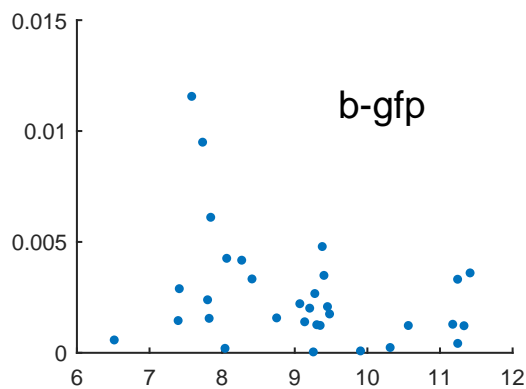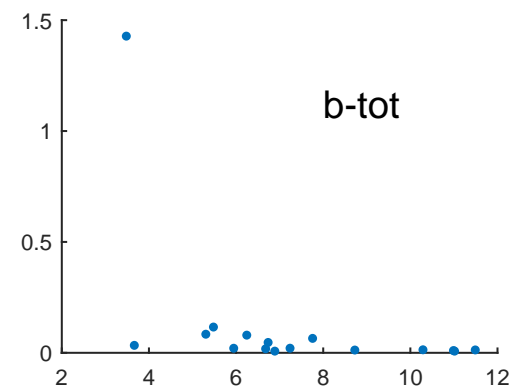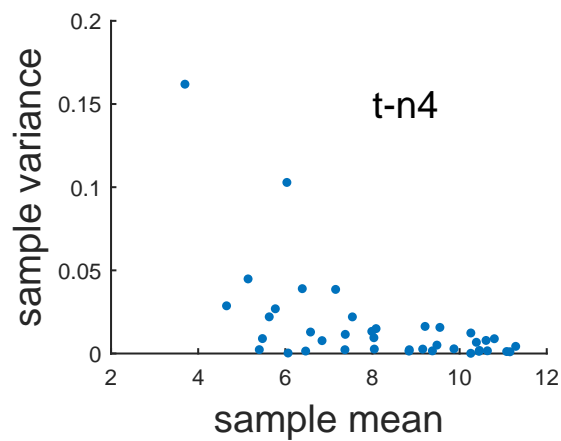

Supplement: S1 Fig — Plots show means and variances of log-transformed data for each of the datasets. Variance can span several orders of magnitude and it depends on the mean. This violates assumptions of LogNrm approach. (PDF) [file pone.0146227.s002.pdf]

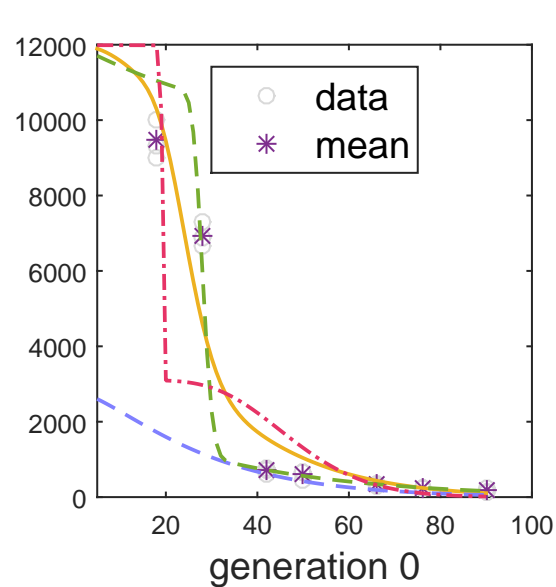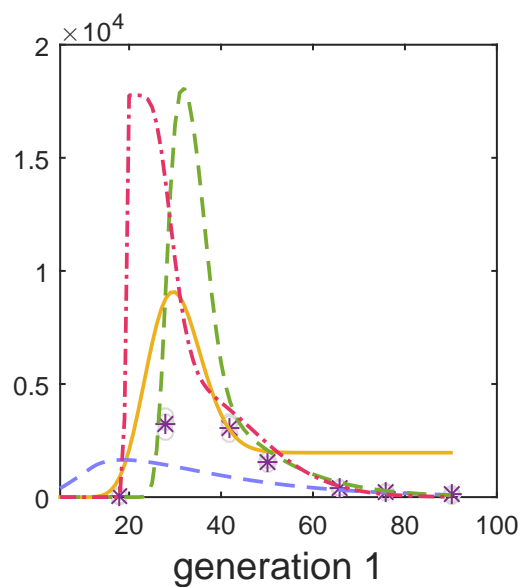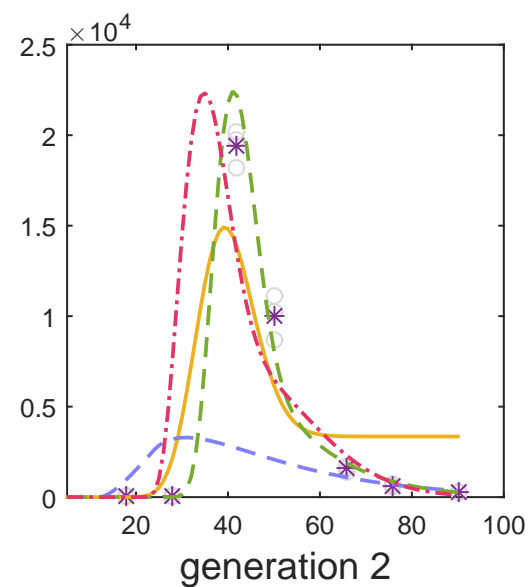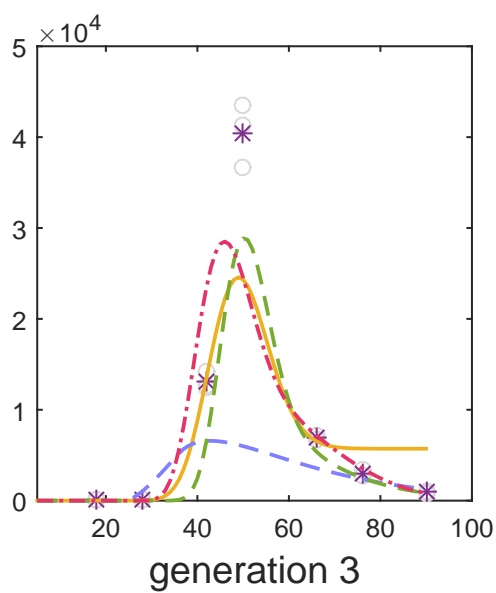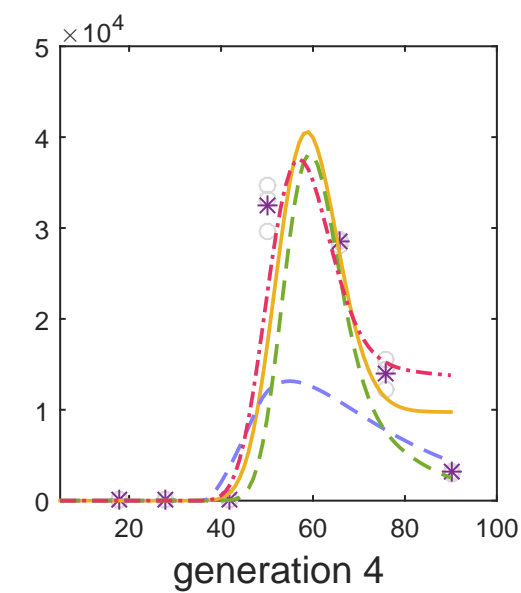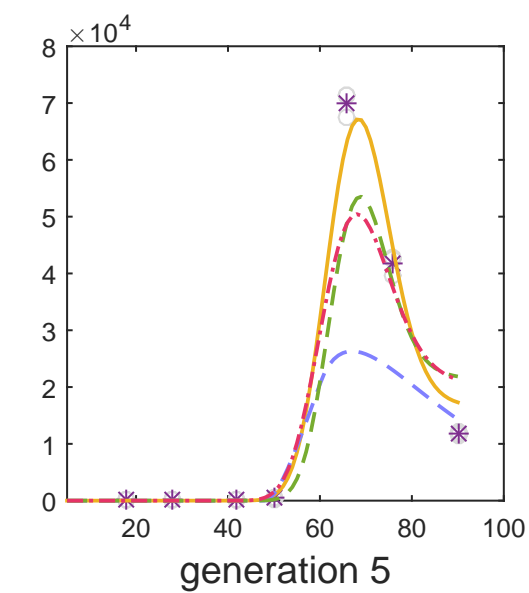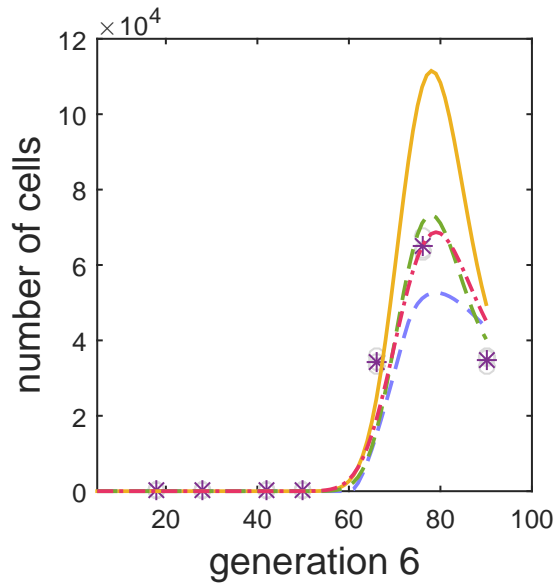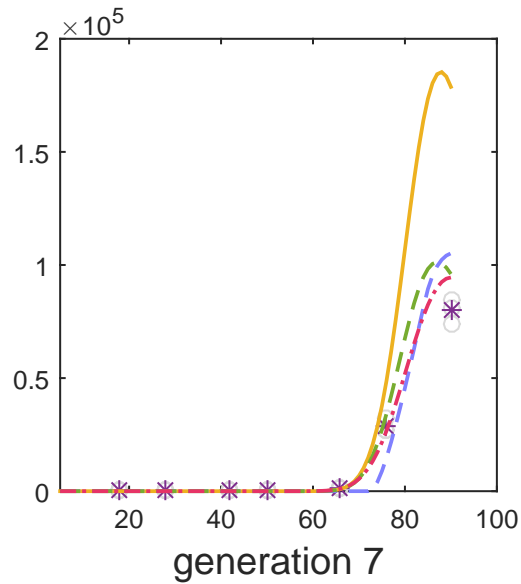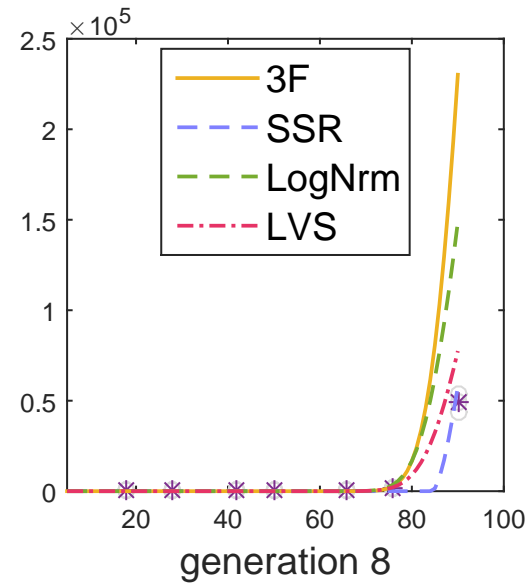

Supplement: S2 Fig — ADMBP model was fitted to t–n4 data using different measurement models. Resulting AICc scores are 2320.7 (3F), 4046.2 (SSR), 4166.8 (LogNrm), and 311694.4 (LVS). For the fit obtained using LogNrm model, AICc was computed using SSR model. (PDF) [file pone.0146227.s003.pdf]

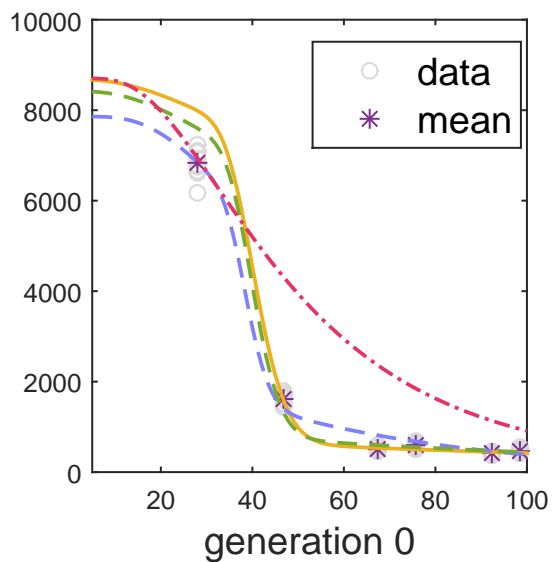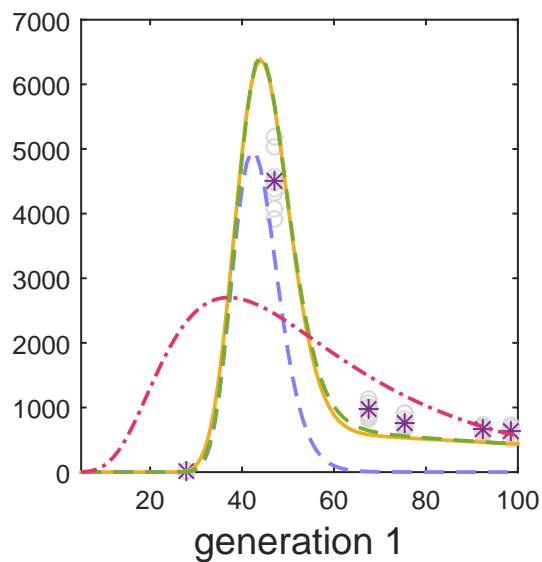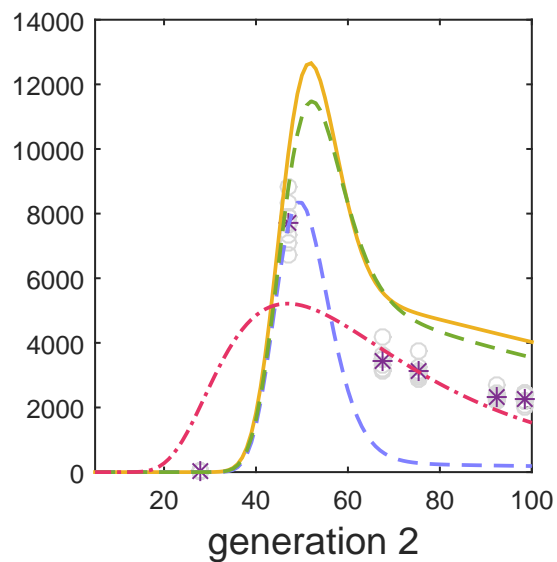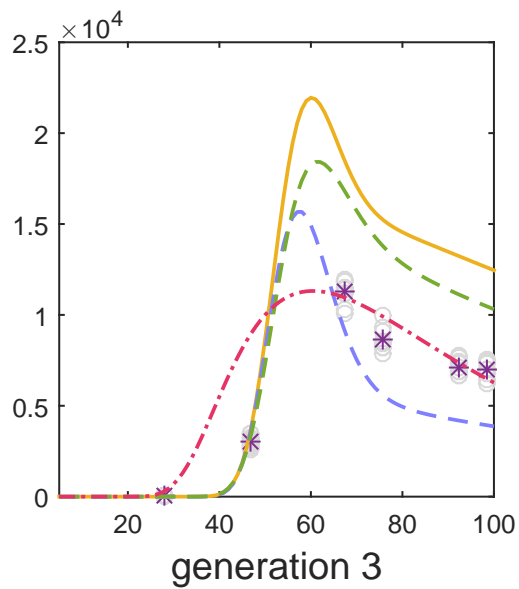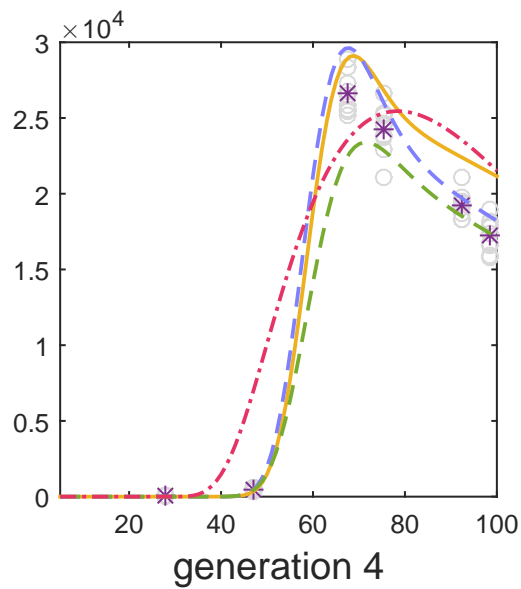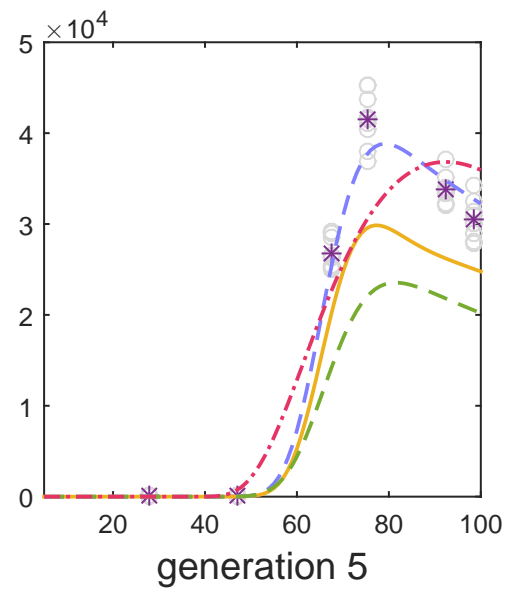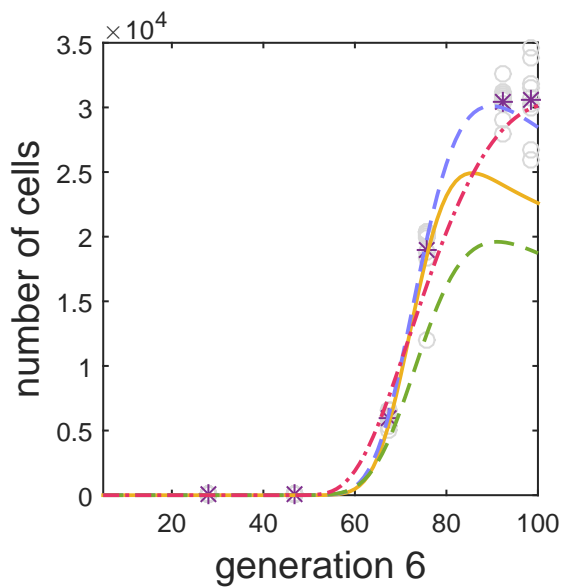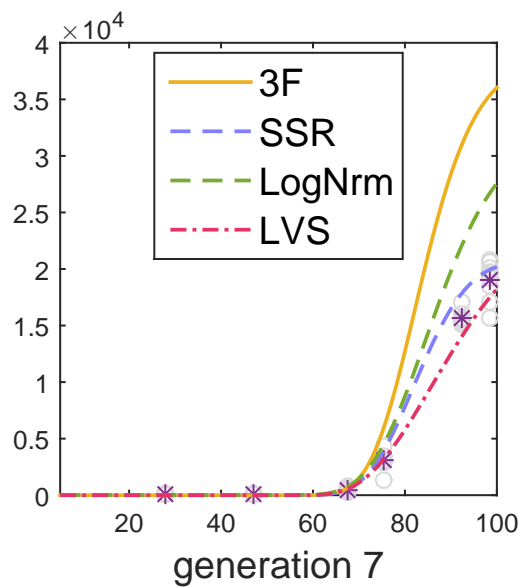

Supplement: S3 Fig — Cyton model was fitted to b–bimko data using different measurement models. Resulting AICc scores are 8238.4 (3F), 10326.0 (SSR), 11602.7 (LogNrm), and 436803.5 (LVS). For the fit obtained using LogNrm model, AICc was computed using SSR model. (PDF) [file pone.0146227.s004.pdf]

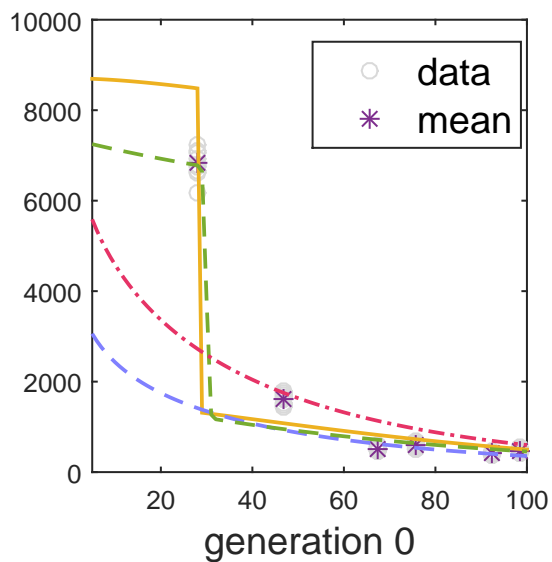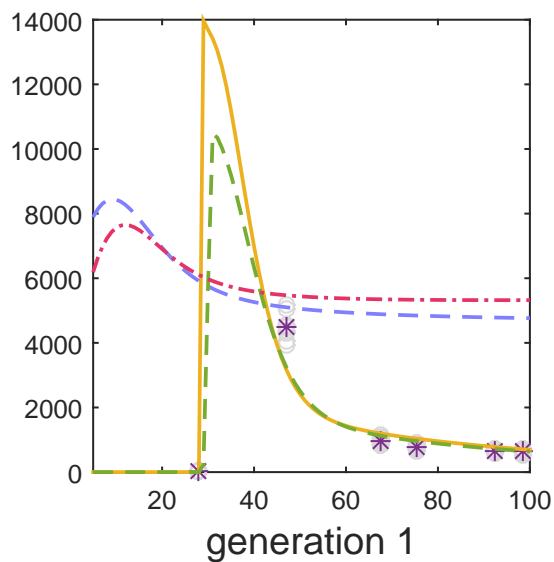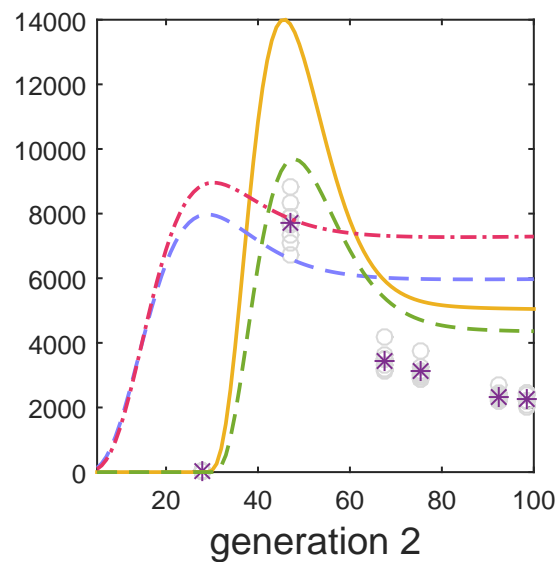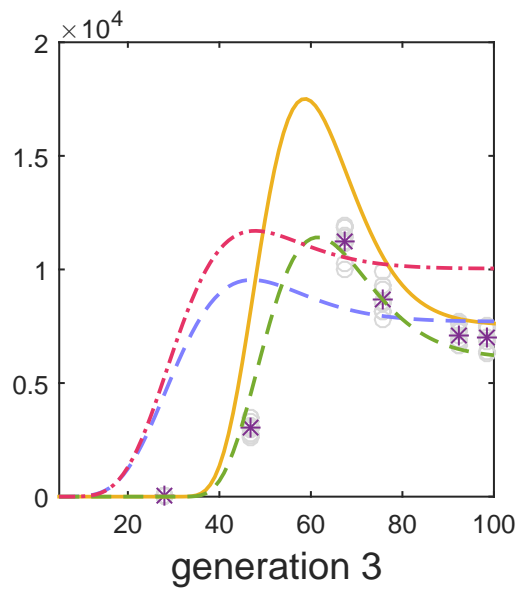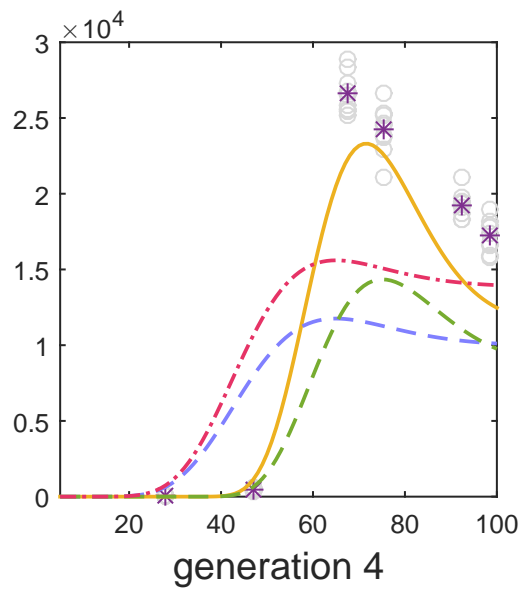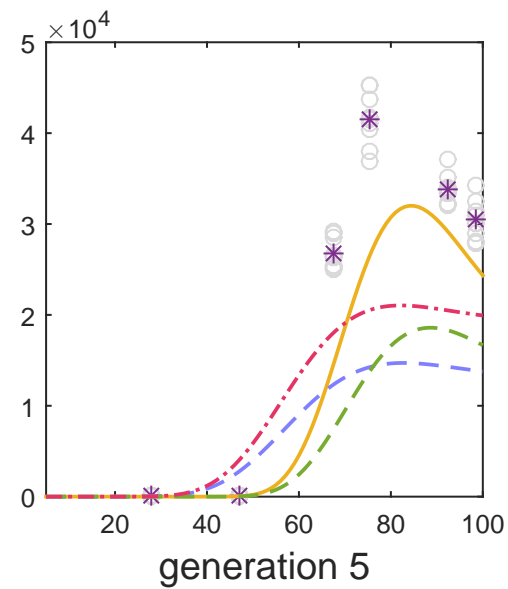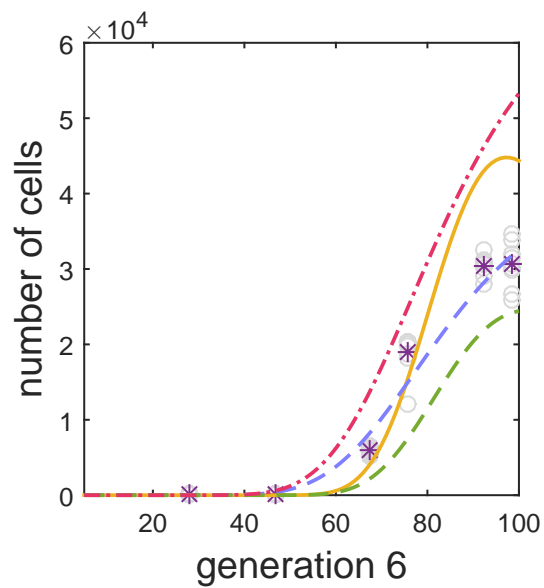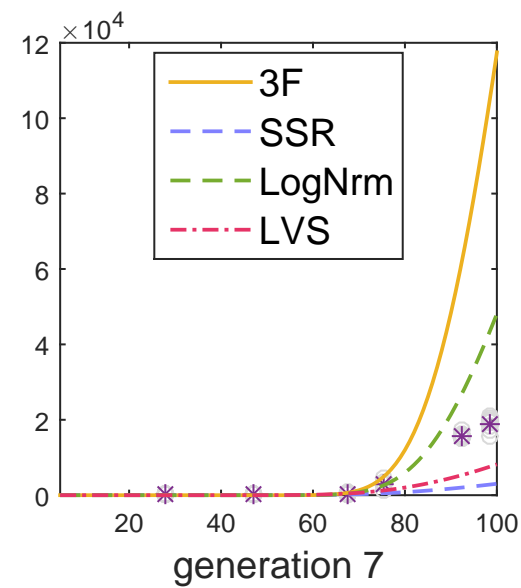

Supplement: S4 Fig — ADMBP model was fitted to b–bimko data using different measurement models. Resulting AICc scores are 9085.5 (3F), 12024.4 (SSR), 13125.6 (LogNrm), and 2674181.0 (LVS). For the fit obtained using LogNrm model, AICc was computed using SSR model. (PDF) [file pone.0146227.s005.pdf]

number of cells

$10^5$   
 $10^4$

15  $\mu\text{g/mL}$

5.6  $\mu\text{g/mL}$

1.9  $\mu\text{g/mL}$

0

20

40

60

80

time, h

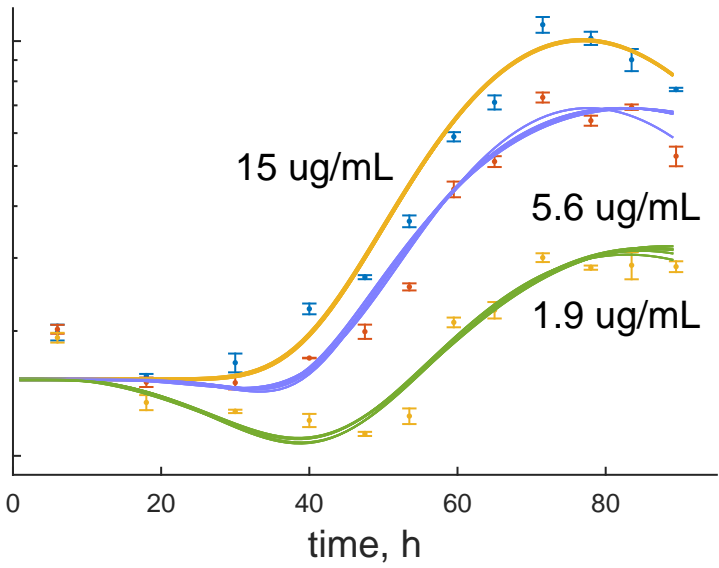

Supplement: S5 Fig — Cyton model was fitted to a representative dataset (in this case, b–lps), and the fitting was repeated several times using different ε ranging from 0.25ε* to 4ε*, where ε* = 50. The choice of ε made little difference to results. Note that this is a multiconcentration dataset, and the fit was performed to all concentrations simultaneously. Further, each time point contains breakdown of the number of cells per generation, but in the figure, only the total number are shown. (PDF) [file pone.0146227.s006.pdf]

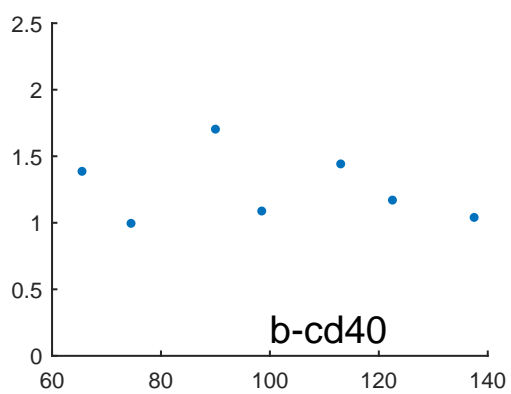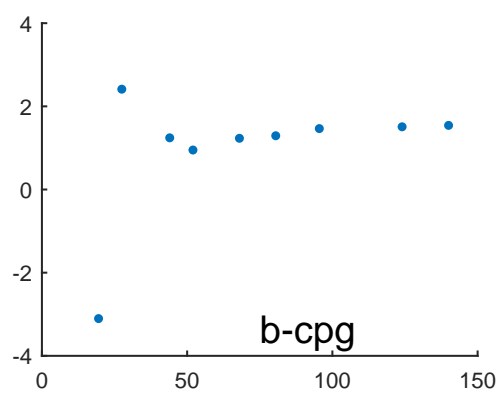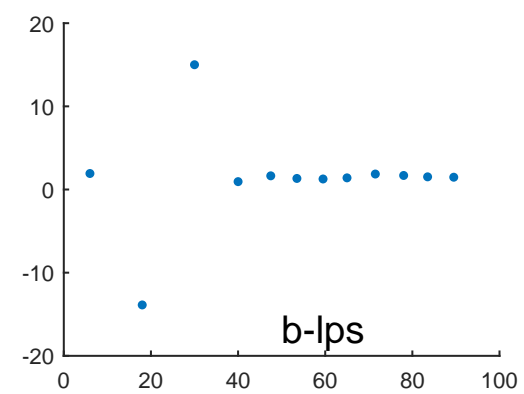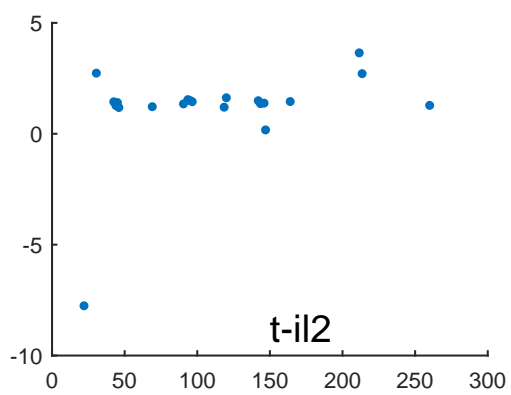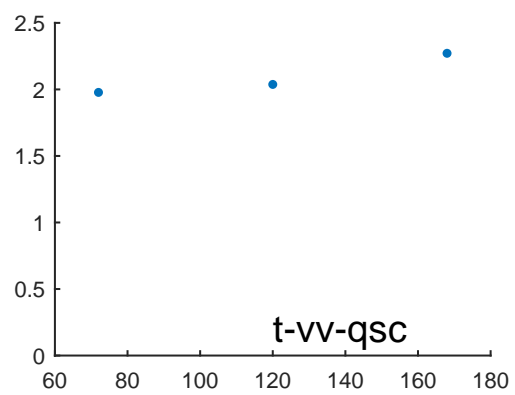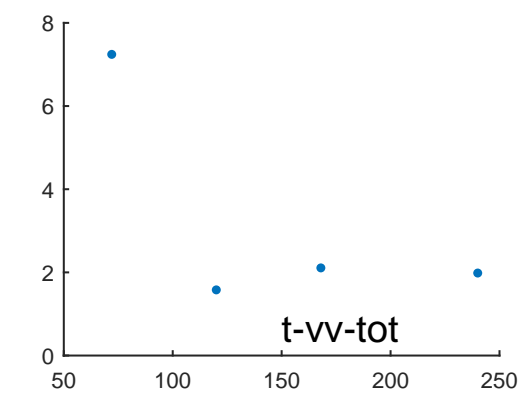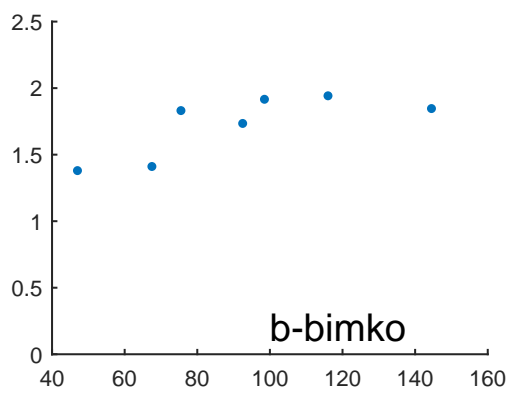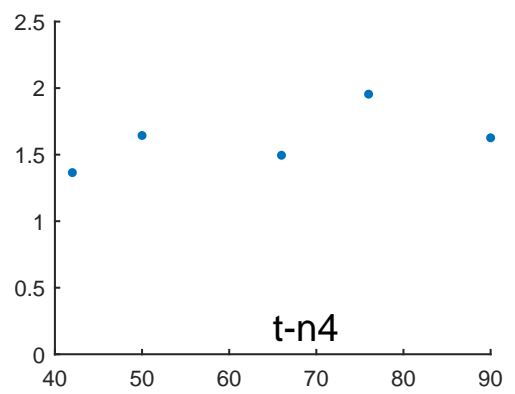

Supplement: S6 Fig — Each point shows the power law exponent estimated from samples from a particular time point. Any given time point comprises several measurement samples (e.g., number of cells in division 1 measured 3 times is one sample; number of cells in division 2 measured 3 times is another sample, etc.). Each sample is then summarized by sample mean and sample variance, a power law relation between mean and variance is fit to data from a single time point, and the exponent is plotted against time. We used only time points with more than 3 samples. Datasets b-gfp and b-tot did not have time points that satisfy this criterion. Overall, there appears to be a slight increasing trend for each dataset, and this observation can be exploited in future measurement models. (PDF) [file pone.0146227.s007.pdf]
