# Supplementary material for: Stochastic Measurement Models for Quantifying Lymphocyte Responses Using Flow Cytometry
Source: PLoS One. 2016 Jan 7;11(1):e0146227. doi: 10.1371/journal.pone.0146227 (PMC4704825; doi:10.1371/journal.pone.0146227)
Supplement: S1 Text — Formal description of some previously proposed measurement models. (PDF) [file pone.0146227.s012.pdf]

S1 Text. Previous Measurement Models

Sum of squared residuals (SSR)

This model was used for model selection using Akaike Information Criterion with a correction (AICc) or Bayesian Information Criterion (BIC) in some previous studies (Hawkins *et al.*, 2013; Miao *et al.*, 2012). The model assumes a multivariate normal distribution of observations, independent measurement vector components, and constant marginal variances, that is, for time point t and component i

$$Z_{t,i}|(\boldsymbol{\theta}^* = \boldsymbol{\theta}) \sim \mathcal{N}(\mu_{t,i}(\boldsymbol{\theta}), \upsilon) \tag{1}$$

Here p components of $\boldsymbol{\mu}$ are predicted by the response model.

Under these assumptions

$$Prob(\mathbb{D}|\boldsymbol{\theta}) = \prod_{t=1}^{d}\prod_{r=1}^{s_t}\prod_{i=1}^{p}\left(\frac{1}{\sqrt{2\pi\upsilon}}\exp\left[-\frac{(z_{t,r,i}-\mu_{t,i})^2}{2\upsilon}\right]\right), \tag{2}$$

where t iterates time points, r iterates replicates (independent runs), and i iterates the components of the measurement vector. The log-likelihood is then

$$\ln\ Prob(\mathbb{D}|\boldsymbol{\theta}) = -\frac{1}{2}\left(n\ln 2\pi + n\ln\upsilon + \frac{1}{\upsilon}s_{ssr}\right) \tag{3}$$

Here $n = p\sum_{t=1}^{d} s_t$, and sum of squared residuals is

$$s_{ssr} \equiv \sum_{t=1}^{d}\sum_{r=1}^{s_t}\sum_{i=1}^{p}(z_{t,r,i}-\mu_{t,i})^2 \tag{4}$$

We have that the derivative with respect to υ is

$$\frac{\partial \ln\ Prob(\mathbb{D}|\boldsymbol{\theta})}{\partial\upsilon} = -\frac{1}{2}\left(\frac{n}{\upsilon} - \frac{s_{ssr}}{\upsilon^2}\right) \tag{5}$$

Setting this to 0 gives $\upsilon^* = s_{ssr}/n$, and plugging this estimate back into the log-likelihood expression yields

$$\ln\ Prob(\mathbb{D}|\boldsymbol{\theta}) = -\frac{n}{2}\left(\ln 2\pi + \ln\frac{s_{ssr}}{n} + 1\right) \tag{6}$$

Computing AICc or BIC require specification of the number of free parameters $k = k_r + k_m$. This number include both the number of the response model parameters, i.e., k_r equals to the number of dimensions of $\boldsymbol{\theta}$, and the number of parameters incurred by the measurement model k_m. In SSR model the incurred parameter is the variance of the measurements, and so $k_m = 1$.

Lognormal measurement distribution (LogNrm)

This model was used for both model fitting and model selection in a previous study (Luzyanina *et al.*, 2013). The model assumes independent log-normally distributed measurement vector components, with a single parameter v which is the variance of the associated normal distribution. Moreover, an intercept c has to be introduced to prevent taking logarithms of zero. Under these assumptions

$$Prob(\mathbb{D}|\boldsymbol{\theta}) = \prod_{t=1}^{d}\prod_{r=1}^{s_t}\prod_{i=1}^{p}\left(\frac{1}{z_{t,r,i}^{(cap)}\sqrt{2\pi v}}\exp\left[-\frac{\left(\ln\left(z_{t,r,i}^{(cap)}\right)-\ln\left(\mu_{t,i}^{(cap)}\right)\right)^2}{2v}\right]\right) \tag{7}$$

Here $z_{t,r,i}^{(cap)} = \max(c, z_{t,r,i})$, $\mu_{t,i}^{(cap)} = \max(c, \mu_{t,i})$, p components of $\boldsymbol{\mu}$ are predicted by the response model, t iterates time points, r iterates replicates (independent runs), and i iterates the components of the measurement vector. The log-likelihood is then

$$\ln\ Prob(\mathbb{D}|\boldsymbol{\theta}) = -\frac{1}{2}\left(n\ln 2\pi + n\ln v + \frac{s_{log}}{v}\right) - \sum_{t=1}^{d}\sum_{i=1}^{p}\sum_{r=1}^{s_t}\ln\left(z_{t,r,i}^{(cap)}\right), \tag{8}$$

where $n = p\sum_{t=1}^{d} s_t$, and sum of squared log residuals is

$$s_{log} \equiv \sum_{t=1}^{d}\sum_{i=1}^{p}\sum_{r=1}^{s_t}\left(\ln\left(z_{t,r,i}^{(cap)}\right)-\ln\left(\mu_{t,i}^{(cap)}\right)\right)^2 \tag{9}$$

We have that the derivative with respect to v is

$$\frac{\partial \ln\ Prob(\mathbb{D}|\boldsymbol{\theta})}{\partial v} = -\frac{1}{2}\left(\frac{n}{v} - \frac{s_{log}}{v^2}\right) \tag{10}$$

Setting this to 0 gives $v^* = s_{log}/n$, and plugging this estimate back into the log-likelihood expression yields

$$\ln\ Prob(\mathbb{D}|\boldsymbol{\theta}) = -\frac{n}{2}\left(\ln 2\pi + \ln\frac{s_{log}}{n} + 1\right) - \sum_{t=1}^{d}\sum_{i=1}^{p}\sum_{r=1}^{s_t}\ln\left(z_{t,r,i}^{(cap)}\right) \tag{11}$$

Computing AICc or BIC require specification of the number of free parameters $k = k_r + k_m$. This number include both the number of the response model

parameters, i.e., k_r equals to the number of dimensions of $\boldsymbol{\theta}$, and the number of parameters incurred by the measurement model k_m. In LogNrm model the incurred parameters are the variance of the log-transformed measurements and capping threshold, and so $k_m = 2$.

Linear variance scaling (LVS)

This model is based on a heuristic objective function used for fitting in a previous study (Hawkins *et al.*, 2013). The model assumes a multivariate normal distribution of observations, independent measurement vector components, and constant marginal variances, that is, for time point t and component i

$$Z_{t,i}|(\boldsymbol{\theta}^* = \boldsymbol{\theta}) \sim \mathcal{N}(\mu_{t,i}(\boldsymbol{\theta}), \upsilon_{t,i}), \tag{12}$$

where p components of $\boldsymbol{\mu}$ are predicted by the response model, and marginal variances follow a linear scaling rule

$$\upsilon_{t,i} = \mu_{t,i} + \varepsilon \tag{13}$$

Here, an intercept ε represents minimum variance of the measurement process.

Under these assumptions

$$Prob(\mathbb{D}|\boldsymbol{\theta}) = \prod_{t=1}^{d} \prod_{r=1}^{s_t} \prod_{i=1}^{p} \left(\frac{1}{\sqrt{2\pi \upsilon_{t,i}}} \exp\left[-\frac{(z_{t,r,i} - \mu_{t,i})^2}{2\upsilon_{t,i}} \right] \right), \tag{14}$$

where t iterates time points, r iterates replicates (independent runs), and i iterates the components of the measurement vector. The log-likelihood is then

$$\ln\ Prob(\mathbb{D}|\boldsymbol{\theta}) = -\frac{1}{2} \left(n \ln 2\pi + \sum_{t=1}^{d} \sum_{i=1}^{p} s_t \ln \upsilon_{t,i} + s_{wssr} \right) \tag{15}$$

Here $n = p \sum_{t=1}^{d} s_t$, and the sum of weighted residuals is

$$s_{wssr} \equiv \sum_{t=1}^{d} \sum_{i=1}^{p} \left(\frac{1}{\upsilon_{t,i}} \sum_{r=1}^{s_t} (z_{t,r,i} - \mu_{t,i})^2 \right), \tag{16}$$

where, $\upsilon_{t,i}$ depends on the proliferation model parameters and needs to be taken into account during the likelihood maximization.

Computing AICc or BIC require specification of the number of free parameters $k = k_r + k_m$. This number include both the number of the response model parameters, i.e., k_r equals to the number of dimensions of $\boldsymbol{\theta}$, and the number of parameters incurred by the measurement model k_m. In LVS model the incurred parameter is the minimum variance threshold, and so $k_m = 1$.

References

Hawkins, Edwin.D., Turner, M.L, Wellard, C.J, Zhou, J.H.S, Dowling, M.R & Hodgkin, P.D (2013) Quantal and graded stimulation of B lymphocytes as alternative strategies for regulating adaptive immune responses. *Nature communications* **4**, 2406 doi:10.1038/ncomms3406.

Luzyanina, Tatyana, Cupovic, Jovana, Ludewig, Burkhard & Bocharov, Gennady (2013) Mathematical models for CFSE labelled lymphocyte dynamics: asymmetry and time-lag in division. *Journal of mathematical biology* doi: 10.1007/s00285-013-0741-z.

Miao, Hongyu, Jin, Xia, Perelson, Alan.S & Wu, Hulin (2012) Evaluation of multitype mathematical models for CFSE-labeling experiment data. *Bulletin of Mathematical Biology* **74**(2), 300–326 doi:10.1007/s11538-011-9668-y.
