# Supplementary material for: Stochastic Measurement Models for Quantifying Lymphocyte Responses Using Flow Cytometry
Source: PLoS One. 2016 Jan 7;11(1):e0146227. doi: 10.1371/journal.pone.0146227 (PMC4704825; doi:10.1371/journal.pone.0146227)
Supplement: S2 Text — Complete formal description of the proposed measurement model and parameter estimation methods. (PDF) [file pone.0146227.s013.pdf]

S2 Text. Description of our Measurement Model

Three factor model

Recall that a *dataset* is a set of samples $\mathbb{D} = \{\mathbb{T}_{(1)}, \ldots, \mathbb{T}_{(d)}\}$, where each sample $\mathbb{T}_{(t)} = \{\boldsymbol{z}_{(t,1)}, \ldots, \boldsymbol{z}_{(t,s_t)}\}$ is taken at a distinct time point, and for each time point, measurements are performed in s_t replicates. Each *measurement* $\boldsymbol{z}_{(t,r)}$ is a p-dimensional vector, where component $z_{(t,r),i}$ denotes the estimated number of cells that belong to *group* i. Examples of groups include cell that divided two times since the beginning of the experiment, cells that have differentiated, or cells that have returned to quiescence. We assume that (a) the measurements for different time points are independent, and (b) the measurements $\boldsymbol{z}_{(t,r)}$ for a given time point t are independent and identically distributed (i.i.d.). Where unambiguous we will focus on a single time point and omit subscript t.

A *lymphocyte response model* is (i) a definition of a parameter space Θ, and (ii) a mapping from Θ into a space of probability distributions, so that the *predicted number of cells* for a particular experiment and a given time point is $X_{\boldsymbol{\theta}} \equiv X|\boldsymbol{\theta}$ where $X_{\boldsymbol{\theta}} \sim \mathcal{F}_\theta$, and $\mathcal{F}_\theta$ is some multidimensional probability distribution. Examples of response models include Cyton model and a branching process-based model with parameters such as mean rates of cell division and death (Hawkins *et al.*, 2007; Yates *et al.*, 2007). In practice, it can be difficult to derive an analytic expression for $\mathcal{F}_\theta$, but we assume that we can compute $E\left[X_{\boldsymbol{\theta}}\right] \equiv \boldsymbol{\mu}\left(\boldsymbol{\theta}\right)$. Further, we note that the response model is not perfect and the *true number of cells* is $(X_{\boldsymbol{\theta}} + \Xi)$, where Ξ denotes *modeling error.*

It is assumed that each experiment (i.e., each particular set of experimental conditions) has a corresponding unknown parameter vector $\boldsymbol{\theta}^*$ that describes the experiment best. Measurement random variable Z can then be expressed using what we call a *three factor (3F) model*

$$Z = X_{\boldsymbol{\theta}^*} + \Xi + \Psi, \tag{1}$$

where Ψ is the discrepancy between the true and measured number of cells due to *experimental error.* Our aim is to estimate parameter vector $\boldsymbol{\theta}^*$, and we take a probabilistic approach where estimation requires computing the *likelihood of parameters* based on measurements from all time points

$$\mathcal{L}(\boldsymbol{\theta}|\mathbb{D}) \equiv Prob(\mathbb{D}|\boldsymbol{\theta}^* = \boldsymbol{\theta}) = \prod_{t=1}^{d}\left(\prod_{r=1}^{s_t} P(Z_{(t)} = \boldsymbol{z}_{(t,r)}|\boldsymbol{\theta}^* = \boldsymbol{\theta})\right) \tag{2}$$

Because of independence of time points we can focus on defining $P(Z = \boldsymbol{z}|\boldsymbol{\theta}^* = \boldsymbol{\theta})$. Note that the above model so far is generic, and the previous models (SSR, LogNrm and LVS) can be viewed as special cases of Equation 2. We diverge from previous models in two major aspects. First, we allow Ψ component to have a non-zero mean. Next, we differ in the choice of shape or covariance matrix of $P(Z = \boldsymbol{z}|\boldsymbol{\theta}^* = \boldsymbol{\theta})$.

Assuming independence of replicates is reasonable, because experimental practices are designed to ensure this assumption as close as possible. For example, if several animals are used for the same time point, either each animal constitutes a replicate or their cells are mixed so that implicit grouping within replicates is avoided. Similarly, a typical well arrangement within a plate attempts to achieve homogeneity in culturing conditions, such as not using plate edges (filling them with medium). Finally, using the Dempster's principle from Section "Covariance selection" in this document would be another way to justify independence. Dempster proposed a method for "filling" unknown elements of the covariance matrix that maximized differential entropy of the resulting distribution, and in our context a diagonal covariance matrix is the best choice according to this principle.

Recall that instead of making assumptions on distributions of components X, Ξ, Ψ, we directly consider the distribution of Z. To this end, we define the shape and the first two moments of the distribution as follows.

Choosing the distribution

It is reasonable to assume that the mean $E\left[Z|\boldsymbol{\theta}^* = \boldsymbol{\theta}\right]$ is finite, and there exists a finite positive-definite covariance matrix $Cov\left[Z|\boldsymbol{\theta}^* = \boldsymbol{\theta}\right]$. We observe that $Z \in \mathbb{R}^{p+}$ and use the following lemma.

Lemma 1. *Among all probability distributions supported on $\mathbb{R}^{p+}$ with the given mean $\boldsymbol{\eta}$ and positive-definite covariance matrix $\boldsymbol{\Sigma}$, a truncated multivariate normal (TMVN) distribution has the maximum differential entropy.*

Proof. Let $\mathcal{Q}$ be a TMVN distribution supported on $\mathbb{R}^{p+}$ with underlying normal parameters $\boldsymbol{\mu}$ and $\boldsymbol{\Sigma}$, where $\boldsymbol{\Sigma}$ is positive definite. The mean and covariance matrix of $\mathcal{Q}$ are denoted by $\boldsymbol{\mu}_t$ and $\boldsymbol{\Sigma}_t$ respectively. Further, let $\mathcal{S}$ be a family of distributions of a random variable supported on $\mathbb{R}^{p+}$ with mean $\boldsymbol{\mu}_t$ and covariance matrix $\boldsymbol{\Sigma}_t$ so that $\mathcal{Q} \in \mathcal{S}$, and let $\mathcal{P} \in \mathcal{S}$ be given.

First, we establish this result:

$$
\begin{aligned}
\int (\mathbf{x}-\boldsymbol{\mu})(\mathbf{x}-\boldsymbol{\mu})^T \,\mathrm{d}\mathcal{P}(\mathbf{x}) &= \int \mathbf{x}\mathbf{x}^T - \mathbf{x}\boldsymbol{\mu}^T - \boldsymbol{\mu}\mathbf{x}^T + \boldsymbol{\mu}\boldsymbol{\mu}^T \,\mathrm{d}\mathcal{P}(\mathbf{x}) \qquad (3)\\
&= \boldsymbol{\Sigma}_t + \boldsymbol{\mu}_t\boldsymbol{\mu}_t^T - \int \mathbf{x}\, d\mathcal{P}(\mathbf{x})\boldsymbol{\mu}^T - \boldsymbol{\mu}\int \mathbf{x}\, d\mathcal{P}(\mathbf{x})^T + \boldsymbol{\mu}\boldsymbol{\mu}^T\\
&= \boldsymbol{\Sigma}_t + \boldsymbol{\mu}_t\boldsymbol{\mu}_t^T - \boldsymbol{\mu}_t\boldsymbol{\mu}^T - \boldsymbol{\mu}\boldsymbol{\mu}_t^T + \boldsymbol{\mu}\boldsymbol{\mu}^T\\
&= \boldsymbol{\Sigma}_t + (\boldsymbol{\mu}_t - \boldsymbol{\mu})(\boldsymbol{\mu}_t - \boldsymbol{\mu})^T,
\end{aligned}
$$

where superscript T denotes matrix transpose.

Let $q(\mathbf{x})$ be the probability density function (PDF) of $\mathcal{Q}$ so that $q(\mathbf{x}) = \frac{1}{\alpha}\exp\{-\frac{1}{2}(\mathbf{x}-\boldsymbol{\mu})^T\boldsymbol{\Sigma}^{-1}(\mathbf{x}-\boldsymbol{\mu})\}$. We now calculate the following expression:

$$\begin{aligned}
-\int \log q(\mathbf{x})\, d\mathcal{P}(\mathbf{x}) &= \log\alpha + \frac{1}{2}\int (\mathbf{x}-\boldsymbol{\mu})^T\boldsymbol{\Sigma}^{-1}(\mathbf{x}-\boldsymbol{\mu})\,\mathrm{d}\mathcal{P}(\mathbf{x}) \qquad (4)\\
&= \log\alpha + \frac{1}{2}\int \mathrm{tr}\left[(\mathbf{x}-\boldsymbol{\mu})^T\boldsymbol{\Sigma}^{-1}(\mathbf{x}-\boldsymbol{\mu})\right]\mathrm{d}\mathcal{P}(\mathbf{x})\\
&= \log\alpha + \frac{1}{2}\int \mathrm{tr}\left[\boldsymbol{\Sigma}^{-1}(\mathbf{x}-\boldsymbol{\mu})(\mathbf{x}-\boldsymbol{\mu})^T\right]\mathrm{d}\mathcal{P}(\mathbf{x})\\
&= \log\alpha + \frac{1}{2}\mathrm{tr}\left[\boldsymbol{\Sigma}^{-1}\int(\mathbf{x}-\boldsymbol{\mu})(\mathbf{x}-\boldsymbol{\mu})^T\,\mathrm{d}\mathcal{P}(\mathbf{x})\right]\\
&= \log\alpha + \frac{1}{2}\mathrm{tr}\left[\boldsymbol{\Sigma}^{-1}\big(\boldsymbol{\Sigma}_t + (\boldsymbol{\mu}_t-\boldsymbol{\mu})(\boldsymbol{\mu}_t-\boldsymbol{\mu})^T\big)\right],
\end{aligned}$$

where tr[...] denotes matrix trace.

This result holds for all distributions in $\mathcal{S}$, so in particular in holds for $\mathcal{Q}$. Therefore, the differential entropy of $\mathcal{Q}$ is given by

$$h(\mathcal{Q}) \equiv -\int \log q(\mathbf{x})\,\mathrm{d}\mathcal{Q}(\mathbf{x}) = \log\alpha + \frac{1}{2}\mathrm{tr}\left[\boldsymbol{\Sigma}^{-1}\big(\boldsymbol{\Sigma}_t + (\boldsymbol{\mu}_t-\boldsymbol{\mu})(\boldsymbol{\mu}_t-\boldsymbol{\mu})^T\big)\right] \quad (5)$$

From this, we see that, for all $\mathcal{P}\in\mathcal{S}$, $-\int \log q(\mathbf{x})\,\mathrm{d}\mathcal{P}(\mathbf{x}) = h(\mathcal{Q})$. In this case, we can apply a well-known result to conclude that $h(\mathcal{P}) \leq h(\mathcal{Q})$ and we have that $\mathcal{Q}$ is the maximum entropy distribution for $\mathcal{S}$.□

We conclude that a TMVN distribution is the safest choice of the distribution *a priori*. Furthermore, we investigate the mean–variance relation in a range of experimental results and find that most of the mass of the measurement distribution tends to reside in $\mathbb{R}^{p+}$ (see main text). In this case, TMVN closely resemble multivariate normal distribution (MVN), and so we propose approximate the distribution of Z with a MVN.

Means of measurements

We now consider expected value of Z and we note that

$$E\left[Z|\boldsymbol{\theta}^* = \boldsymbol{\theta}\right] = \boldsymbol{\mu}\left(\boldsymbol{\theta}^*\right) + \boldsymbol{\delta}, \qquad (6)$$

where $\boldsymbol{\delta}$ is a sum of expected values of modeling and experimental errors. We call this sum is called *measurement offset*. This offset in principle very difficult to express analytically. An expression for $\boldsymbol{\delta}$ would lead to derivation of a perfect mathematical model of lymphocyte responses, but in practice no model gives a perfect description of any real biological process. Therefore, we suggest to keep $\boldsymbol{\delta}$ as parameters that need to be estimated from data.

Covariance selection

Our data suggest that diagonal elements υ_{ii} of covariance matrix of Z can be estimated from the mean as

$$\upsilon_{ii} = \alpha \left(\mu_i \left(\boldsymbol{\theta}\right) + \delta_i\right)^{\beta} \tag{7}$$

(see main text). We emphasize that this is based on an empirical observation, and we do not make any assumptions on whether measurement variance is dominated by the biology or experimental error. Furthermore, it is difficult to estimate non-diagonal covariance elements, because the data usually comprise small sample sizes compared to the number of dimensions (e.g., 3 samples of a 5-dimensional vector). Under these circumstances we suggest to apply Dempster's covariance selection principle which in turn is based on the maximum entropy principle (Dempster, 1972).

According to Dempster, if elements (i, j) of a positive definite covariance matrix $\boldsymbol{\Sigma_\theta}$ can be partitioned into two sets: estimated ($\mathbb{A}$) and unknown ($\mathbb{B}$), then one should use a proxy matrix $\hat{\boldsymbol{\Sigma}}_{\boldsymbol{\theta}}$ that has estimated values in positions $(i, j) \in \mathbb{A}$, and have the other elements set such that the inverse $\hat{\boldsymbol{\Sigma}}_{\boldsymbol{\theta}}^{-1}$ has zeros in positions $(i, j) \in \mathbb{B}$. Dempster has shown that given particular positions and values for the estimated elements, there exist a unique proxy matrix. Moreover, for a MVN distribution, the choice of the proxy matrix maximizes the entropy among all covariance matrices that have estimated values in positions $(i, j) \in \mathbb{A}$. We relate Dempster's principle to our problem with the following lemma.

Lemma 2. *If the set of estimated elements $\mathbb{A}$ comprises elements (i, i) on the main diagonal of a matrix, then according to the above principle, the proxy matrix $\hat{\boldsymbol{\Sigma}}_{\boldsymbol{\theta}}$ is a diagonal matrix.*

Proof. Consider a diagonal proxy matrix $\hat{\boldsymbol{\Sigma}}_{\boldsymbol{\theta}}$ with elements (i, i) set to their estimated values. Then the inverse $\hat{\boldsymbol{\Sigma}}_{\boldsymbol{\theta}}^{-1}$ is also a diagonal matrix, and therefore $\hat{\boldsymbol{\Sigma}}_{\boldsymbol{\theta}}$ is a proper proxy for a diagonal covariance matrix $\boldsymbol{\Sigma_\theta}$ according to Dempster principle. Furthermore, for covariance matrix $\boldsymbol{\Sigma_\theta}$ such a proxy is unique, and therefore for a diagonal covariance matrix $\boldsymbol{\Sigma_\theta}$ the proxy matrix is necessarily diagonal.□

Based on these considerations, we choose a MVN with a diagonal covariance matrix as a model for Z.

Parameter estimation

We now have all necessary components to compute $\mathcal{L}(\boldsymbol{\theta}|\mathbb{D})$, and the simplest approach would be to use maximum likelihood (ML) parameter estimation. The problem is that without a prior distribution on $\boldsymbol{\delta}$, dataset $\mathbb{D}$ can be fit with an arbitrary parameter vector $\boldsymbol{\theta}$, and the discrepancy is then attributed to modeling error $\boldsymbol{\delta}$. In practical scenarios, researchers have certain tolerance to the level

of modeling error. Furthermore, it is clear that fits with smaller values of $\boldsymbol{\delta}$ are preferable. Essentially, the above considerations mean that there is a prior distribution on $\boldsymbol{\delta}$ that needs to be taken into account. We therefore perform parameter estimation using maximum *a posteriori* (MAP) approach

$$Prob\left(\boldsymbol{\theta}, \boldsymbol{\delta} | \mathbb{D}\right) \propto Prob\left(\mathbb{D} | \boldsymbol{\theta}, \boldsymbol{\delta}\right) Prob(\boldsymbol{\delta}) Prob(\boldsymbol{\theta}) \tag{8}$$

Researchers can adopt different priors on $\boldsymbol{\delta}$ and $\boldsymbol{\theta}$ depending on their experience. In our case, we note that it is feasible to assume zero mean and a positive definite covariance matrix for $\boldsymbol{\delta}$. Under these circumstances and without any other knowledge about $\boldsymbol{\delta}$, principle of maximum entropy suggests a MVN with diagonal covariance matrix. Next, we follow previous work and assume that components of $\boldsymbol{\theta}$ are distributed uniformly within user-specified bounds (Hawkins *et al.*, 2013; Marchingo *et al.*, 2014).

We define

$$f_{obj}(\boldsymbol{\theta}, \boldsymbol{\delta}) \equiv \ln\left[Prob\left(\mathbb{D} | \boldsymbol{\theta}, \boldsymbol{\delta}\right) \times Prob(\boldsymbol{\delta})\right], \tag{9}$$

and we aim to find

$$\boldsymbol{\theta}', \boldsymbol{\delta}' = \arg\max \ f_{obj}(\boldsymbol{\theta}, \boldsymbol{\delta}), \tag{10}$$

where $\boldsymbol{\theta}, \boldsymbol{\delta} \in \Theta \times \mathbb{R}^{d \times p}$ are subject to constraint

$$\mu_i\left(\boldsymbol{\theta}\right) + \delta_i > 0 \tag{11}$$

Here Θ is the space of feasible parameters, d is the number of time points in $\mathbb{D}$, and p is the number of dimensions of Z. As discussed

$$Prob\left(\mathbb{D} | \boldsymbol{\theta}, \boldsymbol{\delta}\right) = \prod_{t=1}^{d} \prod_{i=1}^{p} Prob\left(\boldsymbol{z}_{t,i} | \boldsymbol{\theta}, \delta_{t,i}\right), \tag{12}$$

$$Prob\left(\boldsymbol{z}_{t,i} | \boldsymbol{\theta}, \delta_{t,i}\right) = \prod_{r=1}^{s_t} \left(\frac{1}{\sqrt{2\pi \upsilon_{t,i}}} \exp\left[-\frac{\left(z_{t,r,i} - \mu_{t,i}(\boldsymbol{\theta}) - \delta_{t,i}\right)^2}{2\upsilon_{t,i}} \right] \right), \tag{13}$$

$$Prob\left(\boldsymbol{\delta}\right) = \prod_{t=1}^{d} \prod_{i=1}^{p} \left(\frac{1}{\sqrt{2\pi\varepsilon}} \exp\left[-\frac{\delta_{t,i}^2}{2\varepsilon} \right] \right), \tag{14}$$

where $\upsilon_{t,i} = \alpha\left(\mu_{t,i}\left(\boldsymbol{\theta}\right) + \delta_{t,i}\right)^{\beta} > 0$, α and β are estimated from linear regression and $\varepsilon > 0$ is a user-specified value.

Finally, it is convenient to define

$$g(\boldsymbol{\theta}) \equiv \arg \max_{\boldsymbol{\delta} \in \mathbb{V}(\boldsymbol{\theta})} \ f_{obj}(\boldsymbol{\theta}, \boldsymbol{\delta}), \tag{15}$$

$$h_{obj}(\boldsymbol{\theta}) \equiv f_{obj}\left(\boldsymbol{\theta}, g(\boldsymbol{\theta})\right), \tag{16}$$

where $\mathbb{V}(\boldsymbol{\theta})$ is a subspace of $\mathbb{R}^{d \times p}$ defined to ensure $\mu_i\left(\boldsymbol{\theta}\right) + \delta_i \geq 0$, and some properties of these problems are established by the lemma below. We then find MAP parameter estimate by maximizing 16. Convenience comes from the fact, that for a fixed $\boldsymbol{\theta}$, problem 15 reduces to a set of independent one-dimensional problems.

Lemma 3. *In problems 15 and 16, there exist max* $\{f_{obj}(\boldsymbol{\theta},\boldsymbol{\delta})|\boldsymbol{\theta}\in\Theta,\boldsymbol{\delta}\in\mathbb{V}(\boldsymbol{\theta})\}$. *Also if* $\mu_i(\boldsymbol{\theta})$ *is a continuous function of* $\boldsymbol{\theta}$ *then* h_{obj} *is a continuous function of* $\boldsymbol{\theta}$.

Proof. Since $\mu_i(\boldsymbol{\theta})$ is assumed to be a continuous function of $\boldsymbol{\theta}\in\Theta$, and $\mathbb{V}(\boldsymbol{\theta}) = (-\mu_i(\boldsymbol{\theta});\infty)$, we have that $\mathbb{V}(\boldsymbol{\theta})$ is continuous at all $\boldsymbol{\theta}\in\Theta$.

Next we show that there exists a *max* $\{f_{obj}(\boldsymbol{\theta},\boldsymbol{\delta})|\boldsymbol{\theta}\in\Theta,\boldsymbol{\delta}\in\mathbb{V}(\boldsymbol{\theta})\}$. Note that this is a continuous function of $\boldsymbol{\mu}(\boldsymbol{\theta})$ and $\boldsymbol{\delta}$, all components of $\boldsymbol{\mu}(\boldsymbol{\theta})$ are bounded from below and above, and all components of $\boldsymbol{\delta}$ are bounded from below. Consider partial derivatives

$$\frac{\partial f_{obj}}{\partial \delta_{t,i}} = -\frac{1}{\varepsilon}\delta_{t,i} - \frac{1}{2}s_t\beta\left(\mu_{t,i}(\boldsymbol{\theta}) + \delta_{t,i}\right)^{-1} - \frac{1}{2\alpha}\sum_{r=1}^{s_t} T(t,r,i), \tag{17}$$

where

$$\begin{aligned} T(t,r,i) \equiv & -2(z_{t,r,i} - \mu_{t,i}(\boldsymbol{\theta}) - \delta_{t,i})\left(\mu_{t,i}(\boldsymbol{\theta}) + \delta_{t,i}\right)^{-\beta} \\ & -\beta(z_{t,r,i} - \mu_{t,i}(\boldsymbol{\theta}) - \delta_{t,i})^2\left(\mu_{t,i}(\boldsymbol{\theta}) + \delta_{t,i}\right)^{-\beta-1}. \end{aligned} \tag{18}$$

As $\delta_{t,i}\to\infty$ there will be a large positive value $M > |z_{t,r,i}| + |\mu_{t,i}(\boldsymbol{\theta})|$ and when $\delta_{t,i} > M$, we have that $\frac{\partial f_{obj}}{\partial \delta_{t,i}} < 0$. Note that $2-\beta > 0$ for our data (S1 Table). Therefore $f_{obj}(\boldsymbol{\theta},\boldsymbol{\delta})$ is decreasing at large values of $\delta_{t,i}$ and there exist a maximum of $f_{obj}(\boldsymbol{\theta},\boldsymbol{\delta})$.

Finally, we follow a modified proof for the maximum theorem to conclude the following (see for example Ok, 2007). Consider two metric spaces Θ and $\mathbb{R}^{d\times p}$, a correspondence $\mathbb{V}:\Theta\to\mathbb{R}^{d\times p}$, and f_{obj} is a continuous function on $(\Theta\times\mathbb{R}^{d\times p})$. Further, $g(\boldsymbol{\theta}) \equiv \arg\max\{f_{obj}(\boldsymbol{\theta},\boldsymbol{\delta}) : \boldsymbol{\delta}\in\mathbb{V}(\boldsymbol{\theta})\}$ for all $\boldsymbol{\theta}\in\Theta$; and $h_{obj}(\boldsymbol{\theta}) \equiv f_{obj}(\boldsymbol{\theta}, g(\boldsymbol{\theta}))$ for all $\boldsymbol{\theta}\in\Theta$. Then if $\mathbb{V}(\boldsymbol{\theta})$ is continuous at some $\boldsymbol{\theta}\in\Theta$ then h_{obj} is continuous at $\boldsymbol{\theta}$. Here the correspondence $\mathbb{V}$ is not compact, but the existence of maximum is shown above.□

Parameters of 3F model

Recall that we consider a response model (e.g., Cyton) embedded within a measurement model, which is, in this case, 3F. Thus, the parameters of the final model comprise both response model parameters denoted as $\boldsymbol{\theta}$, and parameters of 3F model. The parameters of 3F model are $\delta_{t,i}$ for each time point t and group i (e.g., a group can denote cells in a particular generation), α, β, and ε. Here, $\delta_{t,i}$ are estimated by the fitting procedure described in the previous section. Further, we have assumed that α and β are estimated by the mean-variance model for measurement samples, explained in the main text. However, note that α and β can be estimated directly as parameters that maximize the objective function (posterior probability in Equation 9). It follows that

$$\hat{\alpha} = \frac{1}{n}\sum_{t=1}^{d}\sum_{i=1}^{p}\sum_{r=1}^{s_t}\frac{(z_{t,r,i} - \mu_{t,i}(\boldsymbol{\theta}) - \delta_{t,i})^2}{(\mu_{t,i}(\boldsymbol{\theta}) + \delta_{t,i})^{\beta}} \tag{19}$$

maximizes the objective function. Here n is the number of data points, d is the number of time points, p is the number of groups (dimensionality of measurements), and s_t is the number of replicates for each time point. The optimization can be then restated as minimization of

$$f^*_{obj}(\boldsymbol{\theta}, \boldsymbol{\delta}) = \sum_{t=1}^{d} \sum_{i=1}^{p} \left(\frac{\delta^2_{t,i}}{\varepsilon} + \beta s_t \ln(\mu_{t,i}(\boldsymbol{\theta}) + \delta_{t,i}) \right) + n \ln \left(\sum_{t=1}^{d} \sum_{i=1}^{p} \sum_{r=1}^{s_t} \frac{(z_{t,r,i} - \mu_{t,i}(\boldsymbol{\theta}) - \delta_{t,i})^2}{(\mu_{t,i}(\boldsymbol{\theta}) + \delta_{t,i})^\beta} \right). \tag{20}$$

There appear to be no concise analytical expression for $\hat{\beta}$ that minimizes the above expression, and for the purposes of computational efficiency, we keep β fixed, with its value estimated from the mean-variance model as explained above. Further, to speedup the computations we also estimate α from the mean-variance model, and keep it fixed during model fitting. However, after $\boldsymbol{\theta}$ and $\delta_{t,i}$ are estimated, we update α according to Equation 20. This simplification enables us to find reasonable fits in shorter amount of time compared to full optimization, where α and β vary.

Finally, we note that ε, in principle, *cannot* be estimated from the data. From Equation 20, it is apparent that such an estimate suggests $\varepsilon \to \infty$. Recall, that ε is the variance on Gaussian modeling error, which is an *a priori* tolerance set by a human operator. Modeling error is different from experimental noise, and it captures the fact that a mathematical formulation of the lymphocyte response process (e.g., Cyton model) is never a perfect description of the real process. In our evaluation, we set the default value of 50. Further, note that when a good fit is possible, predicted values $\mu_{t,i}(\boldsymbol{\theta})$ are close to $z_{t,r,i}$, and hence $\delta_{t,i}$ can be kept small. However, in this case, term $\delta^2_{t,i}/\varepsilon$ is also small, and ε does not have a large impact on the function value. Indeed, in our evaluation, we find that fitting is not sensitive to the choice of ε (main text).

Technical discussion

In this work, we consider models that describe lymphocyte response progression embedded within probabilistic measurement models. As most, if not all models in the field of lymphocyte response are parametric(De Boer & Perelson, 2013; Marchingo *et al.*, 2014; Miao *et al.*, 2012), we restrict our scope to parametric response models with associated vector of parameters denoted as $\boldsymbol{\theta}$. At the same time, we do not impose any particular restriction on how components of $\boldsymbol{\theta}$ should be interpreted. For example, there can be a response model that assumes a mixture of two cell populations, and three of the components of $\boldsymbol{\theta}$ can correspond to average division times for each of the populations and the ratio of cells within the mixture. Moreover, this response model can be extended to have an arbitrary number of population with distinct division rates, as long as the entire can be described using a finite number of parameters, e.g., with a parametric distribution for proportions of cell from each population. In that regard, our 3F

model is compatible with a wide array of response models, including those that explicitly model a heterogeneous mixture of cell populations.

Within 3F mode, however, the variance as a function of the mean is described using a single pair of parameters α and β, which reflects the fact that many present day response models assume a homogeneous population. Thus, future research can be directed towards developing a measurement model for heterogeneous mixtures.

In regards to measurement variation, we model biological and experimental noise together using empirically derived power law rule. An alternative approach would be to model biological noise within a response model(Subramanian *et al.*, 2008), and supplement it with a theoretical model of experimental procedure. Indeed, there have been a model for the process of sample selection in a flow cytometer(Banks *et al.*, 2013). However, as we describe in the Introduction of the main text, the entire experimental procedure is more complicated (e.g., it usually includes multiple stages of manual gating), and theoretical characterization of the measurement process is a challenging task. Furthermore, not every response model has an accompanying analysis of variability(De Boer & Perelson, 2013). As such, we believe that the proposed power law model (where power exponent fits to the data) is a reasonable trade-off that provides a reasonable explanation of the data, and in fact will become a stepping stone and a baseline for developing more elaborated models.

References

Banks, H.T, Kapraun, D.F, Thompson, W.Clayton, Peligero, Cristina, Argilaguet, Jordi & Meyerhans, Andreas (2013) A novel statistical analysis and interpretation of flow cytometry data. *Journal of biological dynamics* **7**(1), 96–132 doi:10.1080/17513758.2013.812753.

De Boer, Rob.J & Perelson, Alan.S (2013) Quantifying T lymphocyte turnover. *Journal of theoretical biology* doi:10.1016/j.jtbi.2012.12.025.

Dempster, AP (1972) Covariance selection. *Biometrics* .

Hawkins, Edwin.D., Turner, M.L, Dowling, M.R, van Gend, C & Hodgkin, P.D (2007) A model of immune regulation as a consequence of randomized lymphocyte division and death times. *Proceedings of the National Academy of Sciences of the United States of America* **104**(12), 5032–7 doi:10.1073/pnas.0700026104.

Hawkins, Edwin.D., Turner, M.L, Wellard, C.J, Zhou, J.H.S, Dowling, M.R & Hodgkin, P.D (2013) Quantal and graded stimulation of B lymphocytes as alternative strategies for regulating adaptive immune responses. *Nature communications* **4**, 2406 doi:10.1038/ncomms3406.

Marchingo, J..M., Kan, A., Sutherland, R..M., Duffy, K..R., Wellard, C..J., Belz, G..T., Lew, A..M., Dowling, M..R., Heinzel, Susanne & Hodgkin,

P..D. (2014) Antigen affinity, costimulation, and cytokine inputs sum linearly to amplify T cell expansion. *Science* **346**(6213), 1123–1127 doi: 10.1126/science.1260044.

Miao, Hongyu, Jin, Xia, Perelson, Alan.S & Wu, Hulin (2012) Evaluation of multitype mathematical models for CFSE-labeling experiment data. *Bulletin of Mathematical Biology* **74**(2), 300–326 doi:10.1007/s11538-011-9668-y.

Ok, Efe.A. (2007) *Real Analysis with Economic Applications.* Princeton University Press.

Subramanian, V..G., Duffy, K..R., Turner, M.L. & Hodgkin, P..D. (2008) Determining the expected variability of immune responses using the cyton model. *Journal of Mathematical Biology* **56**(6), 861–892 doi:10.1007/s00285-007-0142-2.

Yates, Andrew, Chan, Cliburn, Strid, Jessica, Moon, Simon, Callard, Robin, George, Andrew.J.T & Stark, Jaroslav (2007) Reconstruction of cell population dynamics using CFSE. *BMC bioinformatics* **8**, 196 doi:10.1186/1471-2105-8-196.
