# Supplementary material for: Stochastic Measurement Models for Quantifying Lymphocyte Responses Using Flow Cytometry
Source: PLoS One. 2016 Jan 7;11(1):e0146227. doi: 10.1371/journal.pone.0146227 (PMC4704825; doi:10.1371/journal.pone.0146227)
Supplement: S3 Text — Normality tests suggest that a MVN distribution is a reasonable approximation for the distribution of measurements. Unless otherwise stated, p-values are given for Lilliefors test. (PDF) [file pone.0146227.s014.pdf]

S3 Text. Testing Normality of the Data

Table below provides p-values of single-dimensional and multivariate normality tests. Recall that each dataset comprises independent samples from different time points or conditions, where each sample is single- or multidimensional and each dimension corresponds to an estimated cell number in a particular group, such as “cells in generation three” or “differentiated cells”. Normality tests were only applied to samples that contained more than three measurements, and such samples were found in four datasets. Lilliefors statistic was used for single-dimensional normality tests, while Mardia, Small’s Omnibus, Royston, and Henze-Zirkler (HZ) statistics were used for testing multivariate normality. A review of these multivariate normality tests can be found in (Wang, 2013). We conclude that with the exception of one sample, the data is consistent with the normality assumption. This conclusion can be made if one adopts significance level threshold of 0.01, which is also commonly used in addition to 0.05.

Dataset	Samples	P-values			
t–il2	1 single-dimensional sample with 6 points	0.442			
t–vv–qsc	24 single-dimensional samples with 5 points each	all 24 p-values in no particular order			
		0.500	0.256	0.140	0.500
		0.340	0.302	0.025	0.388
		0.113	0.500	0.408	0.500
		0.389	0.224	0.182	0.500
		0.170	0.500	0.010	0.130
		0.010	0.128	0.316	0.500
t–vv–tot	12 single-dimensional samples with 5 points each	all 12 p-values in no particular order			
		0.500	0.201	0.049	0.407
		0.500	0.500	0.309	0.500
		0.290	0.500	0.500	0.500
b–bimko	a single-dimensional sample with 9 points	0.500			
	one 6−dimensional sample with 9 points	**Mardia**	**Small**	**Royston**	**HZ**
		0.868	0.765	0.970	0.774
	six 8−dimensional samples with 9 points each	0.994	0.206	0.128	0.902
		0.994	0.000	0.000	0.902
		0.994	0.022	0.239	0.902
		0.994	0.322	0.761	0.902
		0.994	0.055	0.215	0.902
		0.994	0.077	0.136	0.902

References

Wang, Chun.Chao (2013) A MATLAB package for multivariate normality test. *Journal of Statistical Computation and Simulation* (August 2014), 1–23 doi: 10.1080/00949655.2013.808638.
